# Genome features of a novel hydrocarbonoclastic *Chryseobacterium oranimense* strain and its comparison to bacterial oil-degraders and to other *C. oranimense* strains

**DOI:** 10.1093/dnares/dsad025

**Published:** 2023-11-11

**Authors:** Amanda Christine Ramdass, Sephra Nalini Rampersad

**Affiliations:** Biochemistry Research Lab (Rm216), Department of Life Sciences, Faculty of Science and Technology, The University of the West Indies, St. Augustine, Trinidad and Tobago, West Indies; Biochemistry Research Lab (Rm216), Department of Life Sciences, Faculty of Science and Technology, The University of the West Indies, St. Augustine, Trinidad and Tobago, West Indies

**Keywords:** adaptation, bioremediation, *Chryseobacterium oranimense*, comparative genomics, petroleum

## Abstract

For the first time, we report the whole genome sequence of a hydrocarbonoclastic *Chryseobacterium oranimense* strain isolated from Trinidad and Tobago (COTT) and its genes involved in the biotransformation of hydrocarbons and xenobiotics through functional annotation. The assembly consisted of 11 contigs with 2,794 predicted protein-coding genes which included a diverse group of gene families involved in aliphatic and polycyclic hydrocarbon degradation. Comparative genomic analyses with 18 crude-oil degrading bacteria in addition to two *C. oranimense* strains not associated with oil were carried out. The data revealed important differences in terms of annotated genes involved in the hydrocarbon degradation process that may explain the molecular mechanisms of hydrocarbon and xenobiotic biotransformation. Notably, many gene families were expanded to explain COTT’s competitive ability to manage habitat-specific stressors. Gene-based evidence of the metabolic potential of COTT supports the application of indigenous microbes for the remediation of polluted terrestrial environments and provides a genomic resource for improving our understanding of how to optimize these characteristics for more effective bioremediation.

## 1. Introduction

The *Chryseobacterium* genus consists of gram-negative bacteria assigned to the family *Weeksellaceae,* phylum *Bacteroidetes*,^1^ of which there are 160 species and two sub-species (http://www.bacterio.net/). Member species of this genus are adapted to survive in a range of ecosystems and three *Chryseobacterium* species have been reported as survivalists in oil-polluted soil, *C. hungaricum*,^2,3^*C. indoltheticum*^4^ and *C. nepalense*.^5^

Recently and for the first time, the isolation and identification of a crude oil-degrading *C. oranimense* strain COTT from chronically polluted soil in Trinidad was reported.^6^ The novelty of the Trinidad *C. oranimense* strain COTT, compared to other *C*. *oranimense* strains, lies in the long-term exposure and evolution of enhanced capabilities for biotransformation of petroleum in an extreme terrestrial environment; one that has been chronically polluted with crude oil as a result of natural oil seeps that have been in existence for more than 100 years.^7^ Given the short generation times of some microbes that allow for up to tens of generations of evolution daily, indigenous microbes can exhibit selective enrichment and undergo genetic modifications, which enable higher degradation rates.^8^ Additionally, artificially introduced microbes have difficulty competing with the pre-existing/indigenous microbial community that have evolved to survive in a particular habitat.^9^

While it is expected that whole genome sequencing of different *Chryseobacterium* strains will reveal both niche-specific and convergent genome features, the COTT strain is hydrocarbonoclastic and it is not known what genomic traits support specific strategies employed in the metabolism and catabolism of hydrocarbons, as well as other xenobiotics.^9^ Additionally, to what degree these unique features are required to sustain long-term survival in naturally occurring oil seeps is also unknown.

The main objective of this study was to characterize the genome of the *C. oranimense* hydrocarbonoclastic strain, COTT. We sequenced, assembled, annotated and characterized the genome of COTT. An inventory of unique genes of the COTT genome compared to the high-quality genomes of 18 other crude oil-degrading bacterial species, and two other strains of *C. oranimense* was also generated. The findings of this study extend the current knowledge about the genome sequence diversity of the *Chryseobacterium* genus as well as intra-specific genome variation of *C. oranimense*. Genome analysis gave new insights into this strain’s capacity to degrade petrogenic and xenobiotic pollutants, in addition to some of its unique mechanisms for coping with different niche-specific stressors. We anticipate that this genome data will additionally provide an invaluable contribution towards the discovery of industrially important biocatalysts.

## 2. Materials and methods

### 2.1. Strain isolation and petroleum utilization

Strain COTT was initially isolated and stored from soil chronically contaminated with crude oil from an abandoned well in the Fyzabad Forest Reserve, south Trinidad. Details of the site, strain isolation, identification, characterization of crude oil utilization (2% crude oil bioassay) and initial identification can be found in Ramdass and Rampersad.^6^

### 2.2. Phylogenetic analysis

The bacterial Phylogenetic Tree Building Service on the BV-BRC (Bacterial and Viral Bioinformatics Resource Center) server (https://www.bv-brc.org/) was used for the construction of a custom phylogenetic tree built from selected genomes which included that of *C. oranimense* and 19 other *Chryseobacterium* species (Additional file 2: Supplementary Table S1). This Codon Tree method selects single-copy BV-BRC PGFams [both the protein (amino acid) and gene (nucleotide) sequences are used for each of the selected genes from the PGFams] and analyzes aligned proteins and coding DNA from single-copy genes using the program RAxML1A. Protein sequences are aligned using MUSCLE^10^ and the nucleotide coding gene sequences are aligned using the Codon_align function of BioPython.^11^ Support values are generated using 100 rounds of the ‘Rapid’ bootstrapping option^12^ of RaxML. The protein sequences of 1,000 genes aligned in MAFFT were used to produce the final tree and included 949,272 nucleotides and 316,424 amino acids.

### 2.3. Growth and DNA isolation

COTT was grown in Reasoner’s 2A agar (R2A) agar (HiMedia Laboratories LLC., West Chester, PA, USA) in the dark at 25°C for 24 to 48 h. The overnight culture was treated with 500–700 μl of TE buffer (10 mM Tris HCl, 1 mM EDTA, pH8; Sigma-Aldrich, St. Louis, MO, USA) and 100 μl of 50 mg/l each of lysozyme and proteinase K (Sigma-Aldrich, St. Louis, MO, USA) then incubated at 37 °C for 2 h in a water bath, with occasional mixing by inversion. Genomic DNA (gDNA) was extracted using the Maxwell 16 Cell DNA Purification Kit (Promega, Madison, WI, USA) according to the manufacturer’s protocol. Verification of sample quality and quantity, and purity was carried out prior to sequencing.

### 2.4. Whole genome sequencing

The genome of COTT was sequenced by Novogene Corporation Inc., Sacramento, CA, USA. Bacterial genome sequencing was performed using high-throughput Illumina technology on HiSeq using the paired-end (PE150) sequencing strategy. High-quality reads were obtained after quality control was performed in-house by Novogene. The software in the bioinformatics analysis pipeline of the whole genome sequencing (WGS) analyses by Novogene can be viewed in Additional file 2: Supplementary Table S2 and a detailed method in Additional file 4: Supplementary Method.

### 2.5. Genome assembly and annotation

Reads were assembled to contigs in Shovill (Galaxy v 1.1.0+galaxy0) using the Spades assembler. The obtained contigs were validated using QUAST (Galaxy v 5.0.2+galaxy3). Annotation was initially done using BV-BRC Annotations using Subsystems Technology tool kit (RASTtk)^13^ and genome data submitted to NCBI. The Integrated Microbial Genomes Expert Review (IMG/ER) (https://img.jgi.doe.gov/cgi-bin/mer/main.cgi) server of the Joint Genome Institute (JGI) was used for expert annotation of the assembled genome and was the primary source for genome predictions and functional analysis. This server is used for functional annotation and curation of microbial genomes of interest prior to release to GenBank.^14^ The detailed annotation system used by IMG/ER can be viewed in Additional file 2: Supplementary Table S3. Proksee (https://proksee.ca/) was used to generate the circular map of the COTT genome.

### 2.6. Genome analysis—COG and KEGG analysis and comparison

The cluster of orthologous groups (COG) and The Kyoto Encyclopedia of Genes and Genomes (KEGG) pathways in the COTT genome were annotated and analysed, using the abundance profile search tool and the statistical analysis tool on JGI IMG/ER. COG and KEGG CDSs of the COTT genome were compared to the three genomes available for *C. oranimense*: DSM 19055, G311 287168971/referred to as G311_A in text and G311 2602041528/referred to as G311_B in text (Additional file 2: Supplementary Table S4). Venn diagrams comparing the unique gene inventories in the *C*. *oranimense* genomes were done using OmicsBox (https://www.biobam.com/omicsbox). Fisher’s exact *t*-test was used to evaluate statistically significant differences in gene abundance in each COG and KEGG category between the COTT strain and other reference genomes deposited in the IMG bacteria genome database. The COTT genome was compared with 18 crude oil degrading specific bacterial genomes which were obtained via the Genomes by Ecosystem search tool on JGI (Additional file 2: Supplementary Table S5).

### 2.7. Antibiotic susceptibility

Antibiotic susceptibility testing (AST) was performed using HiMedia Antimicrobial Sensitivity Test using Single Test Discs according to the manufacture’s guidelines. Zone size was interpreted as per Clinical & Laboratory Standards Institute (CLSI) (https://clsi.org/) and European Committee on Antimicrobial Susceptibility Testing (EUCAST) (http://www.eucast.org). The experiments were performed in duplicate. Subsequently, antimicrobial resistance genes were identified in JGI. antiSMASH was used for rapid genome-wide identification, annotation and analysis of secondary metabolite biosynthesis gene clusters.^15^

## 3. Results and discussion

### 3.1. Phylogenetic analysis

**Figure 1.**
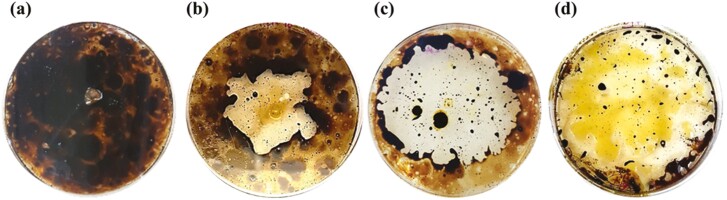
Growth of COTT strain on 3% crude oil-amended Reasoner’s 2A agar after (a) 0 days, (b) 2.5 days, (c) 3.5 days and (d) 5 days.

An axenic COTT strain that was able to grow on 3% crude oil amended media in a previous study was used in this WGS analysis (Fig. 1). The 16S rRNA phylogenetic tree was constructed to show the phylogenetic position of the hydrocarbonoclastic COTT strain and to authenticate the COTT genome assembly (Additional file 1: Supplementary Fig. S1). 16S rRNA sequence comparison confirmed that COTT was most similar to *C. oranimense* type strain H8 (= LMG 24030 = DSM 19055; NR_044168; 100%/99.47% QC/ID)^16^ and *C. oranimense* strain FSB_HA2 (MG322209; 100%/99.05% QC/ID).^17^

**Figure 2.**
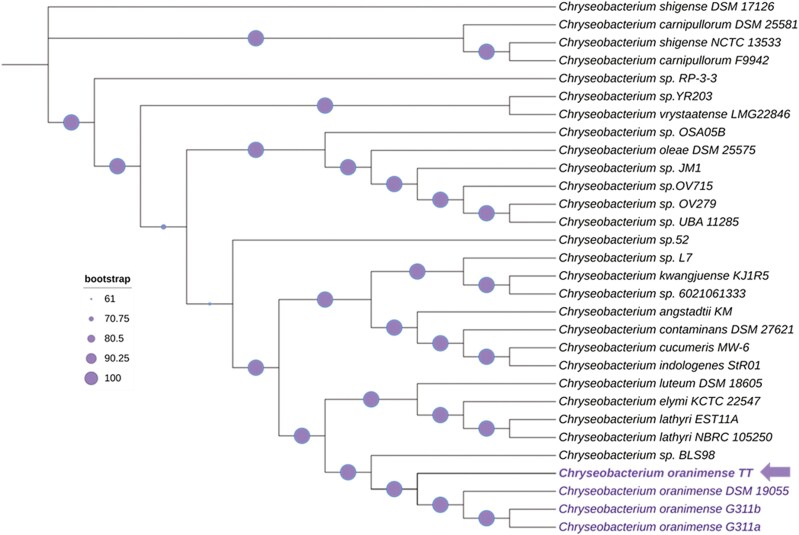
Codon tree of the COTT and 29 related *Chryseobacterium* species. The RaxML tree is based 1,000 CDS alignment on 949,272 nucleotide sequences and 316,424 aligned amino acids. The tree was re-drawn and edited in iTol.

The protein sequences of 1,000 housekeeping genes *C. oranimense* and other species were aligned using the bacterial phylogenetic tree building service in BV-BRC. The tree showed multiple highly supported clades (Fig. 2). Although the COTT strain had the closest similarity to G311 and DSM 19055, COTT was positioned separately within this *C. oranimense* clade; there seemed to be cladogenetic splitting into three distinct branches which may have arisen due to unique morphotype and/or microhabitat relationship within a given community structure.^18^ Cladogenesis as seen here, may indicate some evolutionary advantage to utilize new resources which perhaps, also implies rapid diversification of ecotypes as a trade-off for improved competitive ability in new ecological niches.

### 3.2. Genome project history

A summary of the project and information about the genome sequence is shown in Additional file 2: Supplementary Table S6. A complete report on sequencing data quality control, mapping statistics and SNP, InDel, SV and CNV detection and annotation can be viewed in Additional file 3: Notes and Analysis S1.

### 3.3. Genome features

The genome annotation from JGI IMG/ER was used as the final and primary source for genome predictions and comparisons. As such, detailed data of the annotation data from JGI is presented in the main text and BV-BRC annotation data and analysis can be viewed in Additional file 3: Notes and Analysis S2.

**Figure 3.**
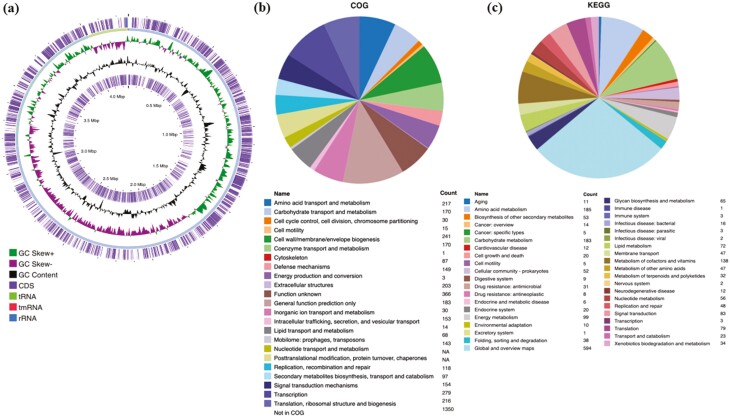
Genome features of *Chryseobacterium oranimense* from Trinidad (COTT). (a) Circular chromosome map of *C. oranimense* TT showing the distribution of CDSs, tRNAs, rRNAs and transfer-messenger RNAs (tmRNAs) and the GC content and skew. From inner centric circle to outer circle: circle 1 represents the scale, circle 2 represents CDS on the reverse strand, circle 3 represents the GC content; circle 4 represents GC skew (+/-); circle 6 represents the CDS on the forward strand and tRNA, rRNA and tmRNA distribution; the blue-green ring (circle 5) represents the backbone of the sequence; this map was generated using Prokesee (https://proksee.ca/). (b) COG statistics by category for the COTT genome. **(c)** KEGG statistics by module for the COTT genome.

The genome features are summarized in Table 1 and Fig. 3. The COTT genome has 11 contigs, a total length of 4,265,502 base pairs (bp), G+C content of 37.91% and a total of 4,040 genes. Biological roles were assigned to 2,794 of the 3,959 predicted protein coding genes (CDS), whereas 1,165 proteins were without a predicted function. 2,690 have CDS with clusters of orthologous groups (COGs) and 936 CDS are connected to KEGG pathways. No plasmids were detected in this strain, similar to the G311 and DSM 19055.^19^ All CDS can be viewed in Additional file 2: Supplementary Table S7. The list of annotated genes and their protein products as assigned to COGs and KEGG Orthology (KO) are presented in Additional file 2: Supplementary Tables S8 and S9, respectively.

**Table 1.** General genome features of COTT

Characteristic	Number	% of Total
DNA, total number of bases	4,265,502	100.00
DNA coding number of bases	3,813,366	89.40
DNA G+C number of bases	1,617,159	37.91
DNA scaffolds	11	100.00
CRISPR Count	1	—
Genes total number	4,040	100.00
Protein coding genes	3,959	98.00
Regulatory and miscellaneous features	2	0.05
RNA genes	79	1.96
rRNA genes	7	0.17
5S rRNA	3	0.07
16S rRNA	3	0.07
23S rRNA	1	0.02
tRNA genes	69	1.71
Other RNA genes	3	0.07
Protein coding genes with function prediction	2,794	69.16
Protein coding genes without function prediction	1,165	28.84
Protein coding genes with enzymes	1,004	24.85
Protein coding genes connected to KEGG pathways	936	23.17
Protein coding genes not connected to KEGG pathways	3,023	74.83
Protein coding genes connected to KEGG Orthology (KO)	1,721	42.60
Protein coding genes connected to MetaCyc pathways	878	21.73
Protein coding genes not connected to MetaCyc pathways	3,081	76.26
Protein coding genes with COGs	2,690	66.58
Protein coding genes with Pfam3	3,009	74.48
Protein coding genes with TIGRfam3	1,007	24.93
Protein coding genes with SMART	693	17.15
Protein coding genes with SUPERFam	3,335	82.55
Protein coding genes with CATH FunFam	2,577	63.79
Protein coding genes in internal clusters	810	20.05
Protein coding genes in chromosomal cassette	4,040	100.00
Chromosomal cassettes	301	-
Protein coding genes coding signal peptides	555	13.74
Protein coding genes coding transmembrane proteins	825	20.42
COG clusters	1,502	55.84
KOG clusters	-	0.00
Pfam clusters	2,005	66.63
TIGRfam clusters	763	75.77

### 3.4. Comparison of COTT to 18 crude oil-degrading bacteria

Comparative analysis was performed to determine the difference in gene abundance in the COTT genome and 18 crude oil-degrading bacteria for which high-quality crude oil-degrading bacterial genomes were available on JGI (Additional file 2: Supplementary Table S10 a-c; abundance of 0 means that a specific gene according to COG ID and COG Name was absent in the species and any number greater than 0 reflects the gene count for that specific gene). Among the COG categories, the COTT genome has a significantly higher mean abundance (Fisher’s Exact *t*-test; *P* < 0.001; *P* < 0.05 Additional file 2: Supplementary Table S11) pertaining to categories such as cell wall/membrane/envelope biogenesis, post-translational modification, protein turnover, chaperones, replication, recombination and repair, and translation, etc. compared to the other 18 genomes. Other COG categories, e.g. responsible for amino acid metabolism, energy production and conversion and lipid metabolism, etc., were significantly lower than the mean average levels in COTT (Fisher’s Exact *t*-test; *P* < 0.001; Additional file 2: Supplementary Table S11).

We then examined gene abundance and found that the COTT genome has 24 characterized CDS in COG, in addition to 16 ‘uncharacterized’ proteins, that were absent from the other 18 genomes (Table 2 and Additional file 2: Supplementary Table S12). There are also 21 proteins present in multiple copies (≥5 copies) in the COTT genome that were present in single or less than 5 copies in the other 18 genomes (Additional file 2: Supplementary Table S13). Statistically (Fisher’s Exact *t*-test; *P* < 0.001; *P* < 0.05), comparative analysis of COG genes between COTT and all 18 genomes showed that there were 28 genes that were significantly different, where 25 were significantly higher in mean abundance in COTT and 3 were significantly lower in COTT compared to the 18 genomes (Additional file 2: Supplementary Table S14).

**Table 2.** Proteins present in COTT but absent in the 18 bacterial crude oil-degrading genomes

COG ID	^1^Protein
COG3669	Alpha-L-fucosidase
COG5184	Alpha-tubulin suppressor and related RCC1 domain-containing proteins
COG1222	ATP-dependent 26S proteasome regulatory subunit
COG4422	Bacteriophage protein gp37
COG2214	Curved DNA-binding protein CbpA, contains a DnaJ-like domain
COG1948	ERCC4-type nuclease
COG4947	Esterase/lipase superfamily enzyme
COG4632	Exopolysaccharide biosynthesis protein
COG1229	Formylmethanofuran dehydrogenase subunit A
COG3968	Glutamine synthetase type III
COG1150	Heterodisulphide reductase, subunit C
COG4068	Predicted nucleic acid-binding protein, contains Zn-ribbon domain
COG4099	Predicted peptidase
COG3432	Predicted transcriptional regulator
COG3586	Predicted transport protein
COG2443	Preprotein translocase subunit Sss1
COG3506	Regulation of enolase protein 1, concanavalin A-like superfamily
COG0724	RNA recognition motif (RRM) domain
COG1603	RNase P/RNase MRP subunit p30
COG1537	Stalled ribosome rescue protein Dom34, pelota family
COG1601	Translation initiation factor 2, beta subunit (eIF-2beta)/eIF-5 N-terminal domain
COG3676	Transposase and inactivated derivatives
COG3033	Tryptophanase
COG5031	Ubiquinone biosynthesis protein Coq4

^1^Protein—there were 16 uncharacterized proteins detected for COTT that were absent in the other 18 hydrocarbonoclastic bacteria strains. COG data for these proteins is shown in Additional file 2, Supplementary Table S12.

The COTT genome has a significantly higher (Fisher’s Exact *t*-test; *P* < 0.001; *P* < 0.05) mean abundance of genes mapped by KEGG (Additional file 2: Supplementary Table S15); notably, proteins of higher abundance were related to multi-drug resistance ^20^. Statistically (Fisher’s Exact *t*-test; *P* < 0.001; *P* < 0.05), comparative analysis of KO genes between COTT and all 18 genomes showed that there were 22 KO genes that were significantly different, all of which were significantly higher in mean abundance in COTT compared to the 18 genomes (Additional file 2: Supplementary Table S16); notably, proteins of higher abundance were related to multi-drug resistance.^20^

### 3.5. Comparison of COTT to other *C. oranimense* genomes

The average nucleotide identity (ANI) score was calculated to be 98.25636% (ANI calculator on JGI IMG/ER) for each of the COTT-G311 and COTT-DSM19055 and for G311-DSM19055 pairwise comparisons.^21^

#### 3.5.1 COG comparisons

**Figure 4.**
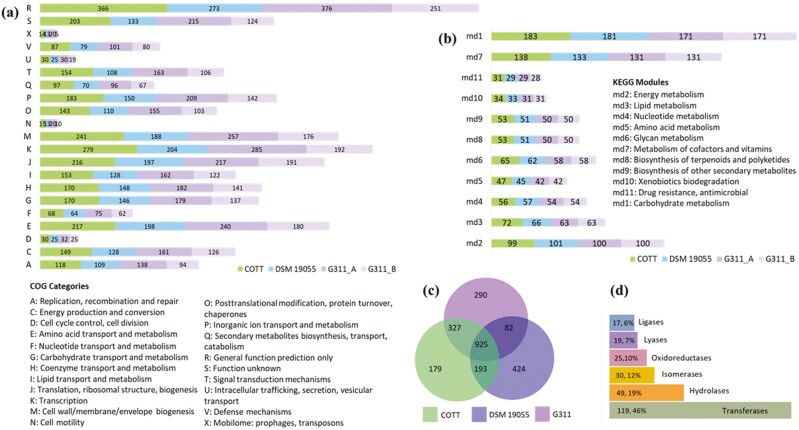
Comparative analysis of gene load according to functional categories among the *Chryseobacterium oranimense* genomes. (a) COG categories and (b) KEGG modules. Each bar indicates the number of genes in COTT, DSM 19055, G311_A, G311_B, respectively. (c) Venn diagram comparing gene inventories in the three *C*. *oranimense* genomes using OmicsBox (https://www.biobam.com/omicsbox). (d) Comparison of COTT genes encoding enzymes of industrial interest.

Analysis of gene abundance in the COG categories between COTT and two *C. oranimense* genomes, DSM 19055 and two assemblies of G311, G311_A (G311 2871689712) and G311_B (G311 2602041528), is shown in Fig. 4(a)–(c) and in Additional file 2: Supplementary Table S17. A comparison of enzymes of industrial interest predicted for COTT is shown in Fig 4(d).

Investigation of the COGs revealed genes that were only found in the COTT genome (Table 3 and Additional file 2: Supplementary Table S18). Fisher’s Exact *t*-test did not reveal any significant differences (*P* < 0.05) in gene abundance according to COG category (Additional file 2: Supplementary Table S19). However, there were higher abundances for genes in COTT compared to the other *C. oranimense* genomes in 9 out of 24 COG categories (Additional file 2: Supplementary Table S19). There were significant differences (*P* < 0.05) in abundance of COG0454 N-acetyltransferase, GNAT superfamily (includes histone acetyltransferase HPA2), COG0477 MFS family permease and COG0457 tetratricopeptide (TPR) repeat (Additional file 2: Supplementary Table S20). The reason may lie in COTT’s need for metabolic self-regulation and survival in soil chronically contaminated with crude oil.^22^

**Table 3.** Novel genes and their abundance (sorted from highest to lowest) found in the COTT genome but absent in the other *C. oranimense* genomes

COG ID	^1^COG name/protein	COTT genome
COG0477	MFS family permease	16
COG0457	Tetratricopeptide (TPR) repeat	16
COG0454	N-acetyltransferase, GNAT superfamily (includes histone acetyltransferase HPA2)	14
COG2771	DNA-binding transcriptional regulator, CsgD family	5
COG2143	Thioredoxin-related protein	4
COG2227	2-Polyprenyl-3-methyl-5-hydroxy-6-metoxy-1,4-benzoquinol methylase	3
COG1277	ABC-type transport system involved in multi-copper enzyme maturation, permease component	3
COG2214	Curved DNA-binding protein CbpA, contains a DnaJ-like domain	3
COG2356	Endonuclease I	3
COG4886	Leucine-rich repeat (LRR) protein	3
COG0500	SAM-dependent methyltransferase	3
COG0764	3-hydroxymyristoyl/3-hydroxydecanoyl-(acyl carrier protein) dehydratase	2
COG2993	Cbb3-type cytochrome oxidase, cytochrome c subunit	2
COG1624	Diadenylate cyclase (c-di-AMP synthetase), DisA_N domain	2
COG2812	DNA polymerase III, gamma/tau subunits	2
COG3391	DNA-binding beta-propeller fold protein YncE	2
COG4782	Esterase/lipase superfamily enzyme	2
COG1145	Ferredoxin	2
COG0545	FKBP-type peptidyl-prolyl cis-trans isomerase	2
COG2329	Heme-degrading monooxygenase HmoA and related ABM domain proteins	2
COG0582	Integrase	2
COG1388	LysM repeat	2
COG0744	Membrane carboxypeptidase (penicillin-binding protein)	2
COG0671	Membrane-associated phospholipid phosphatase	2
COG2185	Methylmalonyl-CoA mutase, C-terminal domain/subunit (cobalamin-binding)	2
COG2169	Methylphosphotriester-DNA–protein-cysteine methyltransferase (N-terminal fragment of Ada), contains Zn-binding and two AraC-type DNA-binding domains	2
COG3118	Negative regulator of GroEL, contains thioredoxin-like and TPR-like domains	2
COG4314	Nitrous oxide reductase accessory protein NosL	2
COG3746	Phosphate-selective porin	2
COG3086	Positive regulator of sigma E activity	2
COG3432	Predicted transcriptional regulator	2
COG1633	Rubrerythrin	2
COG2009	Succinate dehydrogenase/fumarate reductase, cytochrome b subunit	2
COG3829	Transcriptional regulator containing PAS, AAA-type ATPase, and DNA-binding Fis domains	2
COG3706	Two-component response regulator, PleD family, consists of two REC domains and a diguanylate cyclase (GGDEF) domain	2
COG4753	Two-component response regulator, YesN/AraC family, consists of REC and AraC-type DNA-binding domains	2
COG1226	Voltage-gated potassium channel Kch	2
COG0724	RNA recognition motif (RRM) domain	1
COG4335	3-Methyladenine DNA glycosylase AlkC	1
COG1807	4-Amino-4-deoxy-L-arabinose transferase or related glycosyltransferase of PMT family	1
COG2977	4ʹ-phosphopantetheinyl transferase EntD (siderophore biosynthesis)	1
COG0258	5ʹ-3ʹ exonuclease	1
COG0464	AAA+-type ATPase, SpoVK/Ycf46/Vps4 family	1
COG1178	ABC-type Fe3+ transport system, permease component	1
COG2215	ABC-type nickel/cobalt efflux system, permease component RcnA	1
COG1175	ABC-type sugar transport system, permease component	1
COG1118	ABC-type sulphate/molybdate transport systems, ATPase component	1
COG0440	Acetolactate synthase, small subunit	1
COG3830	ACT domain, binds amino acids and other small ligands	1
COG2030	Acyl dehydratase	1
COG2870	ADP-heptose synthase, bifunctional sugar kinase/adenylyltransferase	1
COG0243	Anaerobic selenocysteine-containing dehydrogenase	1
COG4227	Antirestriction protein ArdC	1
COG1166	Arginine decarboxylase (spermidine biosynthesis)	1
COG2730	Aryl-phospho-beta-D-glucosidase BglC, GH1 family	1
COG1222	ATP-dependent 26S proteasome regulatory subunit	1
COG2127	ATP-dependent Clp protease adapter protein ClpS	1
COG0507	ATP-dependent exoDNAse (exonuclease V), alpha subunit, helicase superfamily I	1
COG3241	Azurin	1
COG2193	Bacterioferritin (cytochrome b1)	1
COG2273	Beta-glucanase, GH16 family	1
COG3427	Carbon monoxide dehydrogenase subunit G	1
COG2514	Catechol-2,3-dioxygenase	1
COG4736	Cbb3-type cytochrome oxidase, subunit 3	1
COG0517	CBS domain	1
COG3448	CBS-domain-containing membrane protein	1
COG3027	Cell division protein ZapA, inhibits GTPase activity of FtsZ	1
COG2201	Chemotaxis response regulator CheB, contains REC and protein-glutamate methylesterase domains	1
COG3979	Chitodextrinase	1
COG1605	Chorismate mutase	1
COG4537	Competence protein ComGC	1
COG3245	Cytochrome c5	1
COG3449	DNA gyrase inhibitor GyrI	1
COG1555	DNA uptake protein ComE and related DNA-binding proteins	1
COG2944	DNA-binding transcriptional regulator YiaG, XRE-type HTH domain	1
COG1758	DNA-directed RNA polymerase, subunit K/omega	1
COG1594	DNA-directed RNA polymerase, subunit M/transcription elongation factor TFIIS	1
COG1996	DNA-directed RNA polymerase, subunit RPC12/RpoP, contains C4-type Zn-finger	1
COG1122	Energy-coupling factor transporter ATP-binding protein EcfA2	1
COG0619	Energy-coupling factor transporter transmembrane protein EcfT	1
COG1948	ERCC4-type nuclease	1
COG5309	Exo-beta-1,3-glucanase, GH17 family	1
COG0248	Exopolyphosphatase/pppGpp-phosphohydrolase	1
COG4632	Exopolysaccharide biosynthesis protein related to N-acetylglucosamine-1-phosphodiester alpha-N-acetyl	1
COG5278	Extracellular (periplasmic) sensor domain CHASE3 (specificity unknown)	1
COG0633	Ferredoxin	1
COG0247	Fe-S oxidoreductase	1
COG1580	Flagellar basal body-associated protein FliL	1
COG1360	Flagellar motor protein MotB	1
COG4965	Flp pilus assembly protein TadB	1
COG0355	FoF1-type ATP synthase, epsilon subunit	1
COG0636	FoF1-type ATP synthase, membrane subunit c/Archaeal/vacuolar-type H+-ATPase, subunit K	1
COG1229	Formylmethanofuran dehydrogenase subunit A	1
COG1089	GDP-D-mannose dehydratase	1
COG4767	Glycopeptide antibiotics resistance protein	1
COG4290	Guanyl-specific ribonuclease Sa	1
COG2770	HAMP domain	1
COG1150	Heterodisulphide reductase, subunit C	1
COG0241	Histidinol phosphatase or a related phosphatase	1
COG1169	Isochorismate synthase EntC	1
COG1878	Kynurenine formamidase	1
COG1876	LD-carboxypeptidase LdcB, LAS superfamily	1
COG1934	Lipopolysaccharide export system protein LptA	1
COG1376	Lipoprotein-anchoring transpeptidase ErfK/SrfK	1
COG1556	L-lactate utilization protein LutC, contains LUD domain	1
COG3765	LPS O-antigen chain length determinant protein, WzzB/FepE family	1
COG0474	Magnesium-transporting ATPase (P-type)	1
COG1585	Membrane protein implicated in regulation of membrane protease activity	1
COG4575	Membrane-anchored ribosome-binding protein, inhibits growth in stationary phase, ElaB/YqjD/DUF883 family	1
COG1480	Membrane-associated HD superfamily phosphohydrolase	1
COG0455	MinD-like ATPase involved in chromosome partitioning or flagellar assembly	1
COG0062	NAD(P)H-hydrate repair enzyme Nnr, NAD(P)H-hydrate epimerase domain	1
COG1034	NADH dehydrogenase/NADH:ubiquinone oxidoreductase 75 kD subunit (chain G)	1
COG2816	NADH pyrophosphatase NudC, Nudix superfamily	1
COG1208	NDP-sugar pyrophosphorylase, includes eIF-2Bgamma, eIF-2Bepsilon, and LPS biosynthesis proteins	1
COG0284	Orotidine-5ʹ-phosphate decarboxylase	1
COG2823	Osmotically inducible protein OsmY, contains BON domain	1
COG3133	Outer membrane lipoprotein SlyB	1
COG4238	Outer membrane murein-binding lipoprotein Lpp	1
COG3121	P pilus assembly protein, chaperone PapD	1
COG1732	Periplasmic glycine betaine/choline-binding (lipo)protein of an ABC-type transport system (osmoprotectant binding protein)	1
COG3678	Periplasmic protein refolding chaperone Spy/CpxP family	1
COG3628	Phage baseplate assembly protein W	1
COG5412	Phage-related protein	1
COG1942	Phenylpyruvate tautomerase PptA, 4-oxalocrotonate tautomerase family	1
COG3540	Phosphodiesterase/alkaline phosphatase D	1
COG2194	Phosphoethanolamine transferase for periplasmic glucans (OPG), alkaline phosphatase superfamily	1
COG2091	Phosphopantetheinyl transferase	1
COG0140	Phosphoribosyl-ATP pyrophosphohydrolase	1
COG0047	Phosphoribosylformylglycinamidine (FGAM) synthase, glutamine amidotransferase domain	1
COG0280	Phosphotransacetylase	1
COG5550	Predicted aspartyl protease	1
COG4997	Predicted house-cleaning non-canonical NTP pyrophosphatase, all-alpha NTP-PPase (MazG) superfamily	1
COG0385	Predicted Na+-dependent transporter	1
COG4068	Predicted nucleic acid-binding protein, contains Zn-ribbon domain	1
COG4099	Predicted peptidase	1
COG3111	Predicted periplasmic protein YdeI with OB-fold, BOF family	1
COG2129	Predicted phosphoesterase, related to the Icc protein	1
COG4696	Predicted phosphohydrolase, Cof family, HAD superfamily	1
COG3178	Predicted phosphotransferase, aminoglycoside/choline kinase (APH/ChoK) family	1
COG4127	Predicted restriction endonuclease, Mrr-cat superfamily	1
COG2888	Predicted RNA-binding protein involved in translation, contains Zn-ribbon domain, DUF1610 family	1
COG5567	Predicted small periplasmic lipoprotein YifL	1
COG0653	Preprotein translocase subunit SecA (ATPase, RNA helicase)	1
COG2443	Preprotein translocase subunit Sss1	1
COG0506	Proline dehydrogenase	1
COG1780	Protein involved in ribonucleotide reduction	1
COG0327	Putative GTP cyclohydrolase 1 type 2, NIF3 family	1
COG0748	Putative heme iron utilization protein	1
COG4783	Putative Zn-dependent protease, contains TPR repeats	1
COG1985	Pyrimidine reductase, riboflavin biosynthesis	1
COG0797	Rare lipoprotein A, peptidoglycan hydrolase digesting ‘naked’ glycans, contains C-terminal SPOR domain	1
COG0330	Regulator of protease activity HflC, stomatin/prohibitin superfamily	1
COG0230	Ribosomal protein L34	1
COG0828	Ribosomal protein S21	1
COG1660	RNase adaptor protein for sRNA GlmZ degradation, contains a P-loop ATPase domain	1
COG0594	RNase P protein component	1
COG1603	RNase P/RNase MRP subunit p30	1
COG1592	Rubrerythrin	1
COG1748	Saccharopine dehydrogenase, NADP-dependent	1
COG0515	Serine/threonine protein kinase	1
COG0631	Serine/threonine protein phosphatase PrpC	1
COG3858	Spore germination protein YaaH	1
COG1537	Stalled ribosome rescue protein Dom34, pelota family	1
COG2104	Sulphur carrier protein ThiS (thiamine biosynthesis)	1
COG1838	Tartrate dehydratase beta subunit/Fumarate hydratase class I, C-terminal domain	1
COG0510	Thiamine kinase and related kinases	1
COG3320	Thioester reductase domain of alpha aminoadipate reductase Lys2 and NRPSs	1
COG4115	Toxin component of the Txe-Axe toxin-antitoxin module, Txe/YoeB family	1
COG0781	Transcription termination factor NusB	1
COG4977	Transcriptional regulator GlxA family contains an amidase domain and an AraC-type DNA-binding HTH domain	1
COG3070	Transcriptional regulator of competence genes, TfoX/Sxy family	1
COG1601	Translation initiation factor 2, beta subunit (eIF-2beta)/eIF-5 N-terminal domain	1
COG3415	Transposase	1
COG3677	Transposase	1
COG3676	Transposase and inactivated derivatives	1
COG0130	tRNA U55 pseudouridine synthase TruB, may also work on U342 of tmRNA	1
COG3920	Two-component sensor histidine kinase, HisKA and HATPase domains	1
COG2165	Type II secretory pathway, pseudopilin PulG	1
COG5031	Ubiquinone biosynthesis protein Coq4	1

^1^COG name/protein—there were 57 uncharacterized proteins detected for COTT that were absent in the other *C. oranimense* strains. COG data for these proteins is shown in Additional file 2: Supplementary Table S18.

#### 3.5.2 KEGG comparisons

Genes associated with functional KEGG categories were also examined (Fig. 4(b) and Additional file 2: Supplementary Table S21) and it was found that differences in gene abundance according to KEGG category and KO function were not significant (Additional file 2: Supplementary Table S22 and S23, respectively).

### 3.5.3 Genes only found in the COTT genome

**Figure 5.**
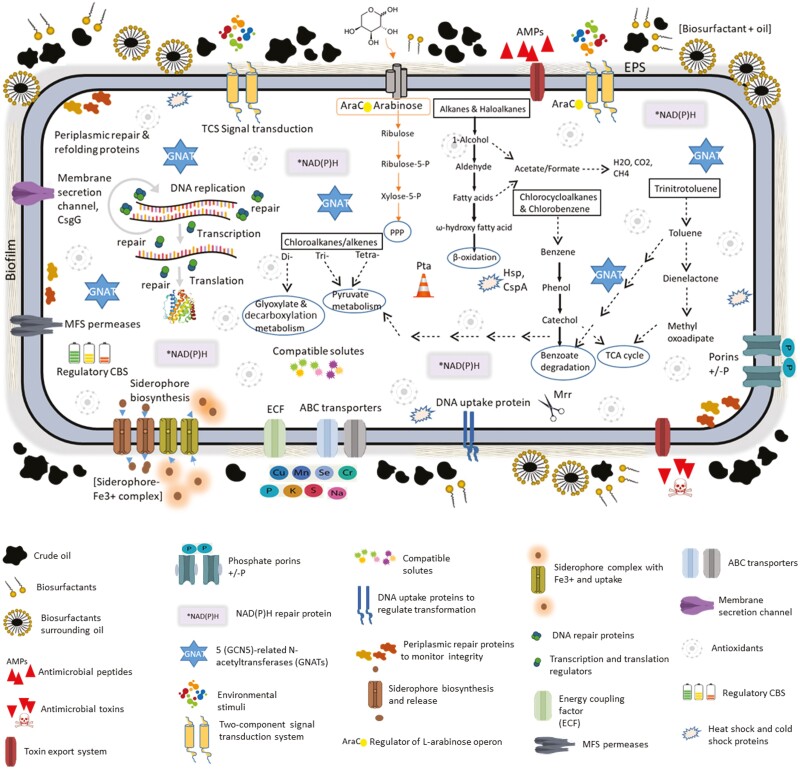
Features of the hydrocarbonoclastic COTT strain. Predicted catabolic pathways (text in oval with blue outline) for bio-transformation of petrogenic compounds (text in boxes with black outline) are indicated by the dashed arrows. Export or import of solutes is designated by the direction of the arrow at the extracellular and cytoplasmic regions of the transporter, respectively. Genome-based model of the cellular metabolism and catabolism including biosurfactant production, toxin export systems, GCN5-related N-acetyltransferases (GNAT) involved in acetylation reactions, e.g., xenobiotic metabolism, two-component systems for bacterial acclimation, CBS domain-containing proteins to sense cellular energy levels, heat shock proteins (Hsp and CspA) as chaperones in correct folding and refolding of proteins and in modulating responses to temperature changes, NAD(P)H-hydrate (*NAD(P)H) repair enzyme prevents hydration of NAD(P)H to NAD(P)HX which inhibits the activity of several dehydrogenases including those involved in xenobiotic metabolism, export and import systems, phosphate-selective porins for aerobic and denitrifying rates of oil degradation, periplasmic repair proteins, membrane secretion channels, siderophore production and export for iron uptake under iron-limiting conditions of crude oil-contaminated soil, antioxidants, DNA repair and uptake proteins, energy-coupling factor (ECF) a unique group of ATP-binding cassette (ABC) transporters for micronutrient uptake, MFS superfamily permeases (uniporters, symporters or antiporters), phosphotransacetylase proteins (Pta) to prevent accumulation of pyruvate/acetyl-CoA where pyruvate serves as an intermediate of central metabolism, AraC protein for relieving sugar-phosphate stress and interaction with histidine kinase sensors in signal transduction in TCS, antimicrobial peptides (AMPs) to inhibit/cell kill bacterial competitors, extracellular polymeric substances (EPS) and biofilm functions.

Bacteria involved in xenobiotic degradation have robust bio-catalytic systems that allow them to function as microbial factories.^23^ The ability of COTT to survive in its environment was predicted based on its unique gene inventories (Fig. 5, Table 3 and Additional file 2: Supplementary Table S18). Genes found in COTT but not in the other *Chryseobacterium* genomes according to the COG category are general function prediction only—1; replication, recombination and repair—6; post-translational modification, protein turnover, chaperones—1; inorganic ion transport and metabolism—3; carbohydrate transport and metabolism—3; coenzyme transport and metabolism—5; cell wall/membrane/envelope biogenesis—2; amino acid transport and metabolism—2; nucleotide transport and metabolism—2; mobilome: prophages, transposons—2; function unknown—8 (Additional file 3: Notes and Analysis S3—Table N12 contains a description of each of the above functions). These gene-based predictions suggest specific areas for functional validation.

### 3.6. Hydrocarbon degradation

#### 3.6.1 Xenobiotic and petrogenic hydrocarbon degradation in COTT

The COTT genome specifies 34 genes encoding proteins associated with xenobiotic biodegradation and metabolism (Additional file 2: Supplementary Table S24) including those associated with benzoate degradation, chloroalkane and chloroalkene degradation, chlorocyclohexane and chlorobenzene degradation, ethylbenzene degradation, metabolism of xenobiotics by cytochrome P450, naphthalene degradation, nitrotoluene degradation, steroid degradation and styrene degradation. Genes encoding enzymes associated with non-specific alkane metabolism were also detected in the COTT genome (Additional file 2: Supplementary Table S25).

Bacteria are known to engage in ring cleavage in the oxidative bio-transformation of crude oil.^24^ The COTT genome encodes numerous proteins involved in the degradation of aromatic compounds: in the phenylpropionate degradation pathway, 2-hydroxy-6-oxonona-2,4-dienedioate hydrolase (*mhp*C) similar to the petroleum oil-degrader *Pseudomonas aeruginosa*^25^; alcohol dehydrogenase (*adh*) and propanol-preferring alcohol dehydrogenase (*adh*P) that act upon aliphatic alcohols, xenobiotic aromatic and aliphatic hydroxyls via similar pathways^26^; gluconolactonase (*gnl*, RGN) that is involved in the degradation of cyclohexanols which are moderately resistant to biodegradation.^27^ 2-Haloacid dehalogenase was found to be encoded in the COTT genome, which suggests that this strain can potentially degrade and/or detoxify recalcitrant halogenated xenobiotic pollutants.^28,29^ Novel sources of haloacid dehalogenase may offer biotechnological applications ranging from waste treatment to synthesis of stereoisomers^30^ and bioremediation.^31^Additional file 2: Supplementary Table S24 contains this data.

Members of the NAD(P)H:FMN oxidoreductase family are thought to participate in oxidative stress responses and in the degradation of xenobiotic compounds including a wide array of nitroalkanes and nitroaromatics.^32^ Evidence of ­2,4,6-trinitrotoluene (TNT) degradation via an anaerobic pathway has been revealed for the COTT strain (http://eawag-bbd.ethz.ch/tnt2/tnt2_map.html). It is possible that COTT adopted this anaerobic mechanism as its genome possesses genes encoding Fe-S oxidoreductase, sulphite reductase, alpha subunit (flavoprotein) and sulphite reductase, beta subunit (haemoprotein), carbon monoxide dehydrogenase and two genes encoding dienelactone hydrolase for anaerobic TNT degradation. Additional file 2: Supplementary Tables S8 and S24 contain this data.

#### 3.6.2 Mono- and di-oxygenases for aromatic degradation

The degradation of aromatic hydrocarbons requires ring activation which is catalyzed by monooxygenases or dioxygenases/dehydrogenases.^33^ Genes encoding nitronate monooxygenase (*ncd2*, *npd*) were detected in COTT. Detoxification of nitroalkane compounds that are used in industry is of considerable interest.^34^ Another example is cholesterol oxidase which is involved in styrene degradation, and is encoded in the COTT genome. Cholesterol oxidase has an application as biocatalysts in industry.^35^

The COTT genome encodes catechol 2,3-dioxygenase (C23O), which catalyzes the meta (extradiol) ring cleavage of catechol and its alkyl derivatives under aerobic conditions. C23O genes are essential for the degradation of monoaromatic hydrocarbons e.g., BTEX and phenol^36–38^ as well as the degradation of PAHs e.g., naphthalene, phenanthrene^39^ and pyrene.^40,41^ Six quercetin 2,3-dioxygenase genes/redox-sensitive bicupin *YhaK* belonging to the pirin family were detected in COTT and these genes have been implicated as associated with hydrocarbon-pollutant degradation.^42^Additional file 2: Supplementary Tables S8 and S24 contain this data.

In addition, oxygenase-type enzymes required for pollutant detoxification, e.g. catalases (*kat*G *and kat*E) and dye-decolourizing peroxidase (DyP-type peroxidases) were identified in the COTT genome. Porphyrinogen peroxidase (*yfe*X) and chloroperoxidase (*cpo*), which are of industrial interest as biological decolourizers of synthetic dyes were also encoded. Additional file 2: Supplementary Tables S7, S8 and S24 contain this data.

#### 3.6.3 Propanoate metabolism

Propionate, a short chain fatty acid (SCFA), is a major component of subsurface petroleum reservoirs.^43^ Propanoate metabolism was found to be stimulated by the presence of crude oil.^44^ SCFAs produced by hydrocarbonoclastic microbes are involved in biodegradation of petroleum and has been shown to be crucial for petroleum recovery and energy extraction.^43,45,46^ In COTT, the succinate pathway appears to be the dominant pathway for propionate production. Additional file 2: Supplementary Table S24 contains this data.

#### 3.6.4 Lipid metabolism

The COTT genome has 72 lipid metabolism genes (Additional file 2: Supplementary Table S24). Lipid metabolism plays an integral role in glycolipid biosurfactant production and in the formation of the lipopolysaccharide and phospholipid scaffolds of the outer membrane of Gram-negative bacteria.^47^ Notably, the esterase and lipase superfamily are economically important enzymes to a range of industries.^48,49^ The COTT genome contains two genes encoding esterase/lipase (COG4782) which were not found in the DSM 19055 strain and only one copy was found in G311. Additional file 2: Supplementary Table S24 contains this data.

### 3.7. Chaperones in protein folding

Chaperones, also known as heat shock proteins, assist in the correct three-dimensional folding of proteins, acting to stabilize or protect disassembled polypeptides, for normal cell growth; they are stress-induced under conditions of high temperatures and in the presence of pollutants and heavy metals.^50^ As expected, the COTT genome harbours a comparatively higher number of genes coding for chaperones, e.g. chaperone modulatory protein CbpM, heat shock proteins GroEL, GroES, HtpG/Hsp90, HSP20, and DnaK, DnaJ, GrpE. Additional file 2: Supplementary Table S24 contains this data.

### 3.8. Quorum sensing and biofilm formation

Quorum sensing (QS) enables communication and exchange of chemical signals in dense populations of bacteria especially in heavily polluted soil^51,52^ and helps to regulate antibiotic production.^53^ QS output with respect to biofilm formation is advantageous as it provides greater resistance to stress, antimicrobial agents, predation and toxic chemicals.^54^ The removal of xenobiotic compounds, including crude oil and PAHs, by biofilm-forming strains has been reported^55^; biofilms of *Burkholderia* sp. NK8 and *P*. *aeruginosa* PA01 have been reported to be involved in the degradation of chlorinated benzoates; biofilm of *P. stutzeri* T102 is involved in the emulsification of naphthalene^54^ phenanthrene, and pyrene in activated sludge.^56^

Twenty-six genes encoding enzymes that function in QS were detected in the COTT genome. In discriminating the lipid environment during QS to facilitate optimal binding to fulfil specific functions and target certain cell types, COTT has thiol-activated cytolysin (*slo*).^57^ In the family of two-component systems (TCSs), extracellular polysaccharide/exopolysaccharides EspA/wza, EspP and EspB/etk-/wzc, were identified in the COTT genome. These gene products are required for bacterial attachment to solid surfaces, to other bacteria and in biofilm formation.^58^ Thirty-two related biofilm formation genes were detected in the COTT including exopolysaccharide (EPS) genes that are known to provide the three-dimensional structure of the biofilm^59^ (Additional file 2: Supplementary Table S18 and S24). Cells use EPS to navigate into the more nutrient and oxygen-rich regions at the air-liquid interface.^60^

In addition to biofilm formation, genes in the COTT genome such as anthranilate synthase component 1 (*trp*E) and component 2 (*trp*G) can also serve as novel drug targets, offer antibiotic tolerance and virulence.^61,62^ Genes encoding multidrug efflux system outer membrane protein (*opr*M) were detected in the QS pathway for COTT. Resistance-nodulation-division efflux pumps have versatile physiological functions, e.g. multidrug resistance, microbial environmental adaptability, pathogenesis and organic solvent tolerance.^63,64^ In the PAH-degrading strain *P. putida* B6-2, efflux pumps were critical for releasing the toxicity caused by intermediates of PAH degradation.^65^ Although the functions of multidrug resistance efflux pumps (MDREPs) in a clinical sense is well-understood, their roles in the degradation of PAHs require additional characterization.^66^Additional file 2: Supplementary Table S24 contains this data.

### 3.9. Membrane transport

Nutrient uptake and elimination of toxic by-products via transporters, e.g., ABC transporters, are essential to microbial survival in polluted environments.^67^ The genome of COTT has a total of 27 genes that encode various ABC transporters (Additional file 2: Supplementary Table S24) in addition to ABC subfamily B multi-drug efflux pump (mdlA, mdlB), which plays a role in lipid A and possibly glycerophospholipid transport.^68^

### 3.10. Secretion systems

Bacterial secretion systems facilitate protein export^69^ attachment to eukaryotic cells, and scavenging for energy resources.^70^ Among the genes responsible for type I, II, V and VI secretion systems present in the COTT genome (Additional file 2: Supplementary Table S24), type VI secretory protein (vgrG), is implicated in (i) bacterial interactions and community structure in a range of environmental niches, (ii) in the ability of many pathogenic bacteria to outcompete rival bacteria, and (iii) in the direct interaction of symbionts with their eukaryotic hosts.^71^

### 3.11. Regulation patterns

Bacteria can sense and respond to environmental stimuli which is important to survival particularly in highly dynamic, competitive niches. There are several recognized classes of sensory signal transduction systems in prokaryotes.^72^ Based on KEGG analysis, 91 CDSs in the COTT genome were classified into signal transduction pathways and of these, 66 were classified into TCSs which consist of two types of signal transducers, a sensor kinase and its cognate response regulator^73^ (Additional file 2: Table S24). The COTT genome contains 18 genes that encode proteins of the OmpR family for TCS responding to phosphate limitation and assimilation, cell envelope protein folding and protein degradation, serine protease, copper/silver efflux, potassium transport, DNA replication, iron acquisition, lantbiotics biosynthesis and immunity and adhesion, autolysis, multidrug resistance and virulence genes. Phosphorous plays a major role in bacterial biological activity in oil reservoirs.^74^ PhoP and PhoR phosphate regulon response and sensor, respectively, were detected in COTT’s genome.

Response regulator YesN is also present in the COTT genome. YesN proteins belong to the AraC/XylS family of transcriptional regulators that control the expression of genes with diverse biological functions.^75^ The apparent redundancy of regulatory genes may indicate a flexible mechanism associated with nitrogen fixation and assimilation.^76^

radC, DNA repair protein, is commonly associated with microorganisms that inhabit harsh environments.^77^ The radC gene was more abundant in the COTT genome than in the other *C*. *oranimense* genomes. The *ato*B gene, which is present in COTT, encodes acetyl-CoA C-acetyltransferase that is involved in short-chain fatty acid metabolism and induces modulation of chemotactic behaviour, as well as other yet undefined responses.^78^ Also detected in this family were C4-dicarboxylate transport proteins DctA (similar to DctP above) and RNA polymerase σ (sigma)-54 factor RpoN.^79^ COTT has four *glo*A genes, which code for a type I glyoxalase, which may serve as an oxygen sensor and regulator in transcription of the Fe-Mo transport operon for nitrogen fixation,^80^ as well as three type II glyoxalases (*glo*B) which may be important to minimize the effects of environmental stressors on protein stability or enzymatic activity in bacteria.^81^

*cbb*_*3*_ oxidases were found in COTT. *cbb*_*3*_ oxidases possess a high affinity for oxygen and are encoded by the tetracistronic *cco*NOQP-1 and *cco*NOQP-2 operons.^82,83^ The enzymatic and transcriptional characteristics of *cbb*_3_ oxidases assist bacteria (e.g. *P. aeruginosa)* to grow in low-oxygen environments.^84,85^ Cytochrome *bd* quinol oxidase was present in COTT and was reported to become active under hypoxic conditions^86,87^ and under high H_2_S concentration.^88^

### 3.12. Nutrient uptake (Fe, S, P, N)

#### 3.12.1 Iron

Most prokaryotes regulate transcription of metal ion-responsive genes to coordinate and regulate metal homeostasis, e.g. ferric uptake regulator, Fur, responds to changes in iron availability.^88^ The *fur/zur* gene is present in the COTT genome, and studies suggest that Fur downregulates the production of iron sequesters, e.g., siderophore biosynthesis.^88^ The ferric enterobactin receptors (fepA, pfeA, iroN, pirA) are also present in the COTT genome. Additionally, COTT has several genes essential to iron uptake into the cytoplasm, including energy transduction genes *exb*B, *exb*D, *ton*B, *tol*Q/*exb*B and the tonB-dependant receptor (TBDR).^89^Additional file 2: Supplementary Table S24 contains this gene inventory.

#### 3.12.2 Sulphur

The COTT genome has 15 genes encoding enzymes involved in sulphur utilization and one sulphate permease encoding gene of the SulP family.^90^ Less attention has been paid to the mechanisms for sulphur (sulphate) uptake from the environment and how it is subsequently assimilated into organic compounds such as cysteine.^91^ There is no data on the mechanism of sulphate uptake in crude oil environments where high concentrations of sulphate exist, as would be the case for the oil-polluted habitat of the COTT strain. The *cys*H,D,N,E,K,G,J,I genes are implicated in the assimilation of sulphur into cysteine in the COTT genome. Additional file 2: Supplementary Table S24 contains this gene inventory.

#### 3.12.3 Phosphorus

phoA,B,H,L,R,B1,P phosphate response regulators belonging to the OmpR family, are present in COTT. These *pho* genes may play a vital role in phosphate/phosphonate transport systems by way of organophosphonate biosynthesis and catabolism. Phosphonates are catabolized by *phn* genes and COTT had one phosphodiesterase (*phn*P), one phosphonoacetate hydrolase (*phn*A) and two PhnB protein (*phn*B) genes.^92,93^ The genome also had phosphate transport system substrate-binding protein (pstS) which encodes a periplasmic phosphate binding protein, exopolyphosphatase (ppx) which, when conditions change and phosphate becomes limiting, allows phosphate to be liberated with no expenditure of energy; ppx has been previously described to be involved in heavy metal resistance and efflux and polyphosphate kinase (ppk), which is present in COTT, is involved in polyphosphate storage.^94^ Phosphate starvation-inducible phoH-like protein (*pho*H/L) was also detected in COTT. GDP-α-D-mannose is a key substrate in glycoprotein formation and is produced by the enzyme mannose-1-phosphate guanylyltransferase (*manC/cpsB*). It is responsible for transferring phosphorus-containing groups and was more abundant on COTT genome than the other *C*. *oranimense* genomes. *cps*B expression is increased by osmotic shock, physiological response for enhanced environmental application.^95^Additional file 2: Supplementary Table S24 contains this gene inventory.

#### 3.12.4 Nitrogen

In the nitrogen regulatory protein C (NtrC) family, three genes *gln*A, *ntr*Y and *ntr*X are involved in nitrogen metabolism when nitrogen availability is low and were found in the COTT genome. Similarly, COTT glutamine synthetase (*gln*A), is involved in glutamate metabolism under limiting conditions.^96^ For symbiotic nitrogen-fixing bacteria, *Azorhizobium caulinodans* and *Azospirillum brasilense ntr*Y gene expression is upregulated in response to hypoxic conditions which in turn signals regulator ntrX to increase expression of nitrogen respiration enzymes.^97^ This system thus functions as a redox sensor. A *drag* gene encoding ADP-ribosyl-[dinitrogen reductase] hydrolase was identified in COTT. *dra*G is a key player in the regulation of nitrogenase activity.^98^ Glutaminase, encoded by *gls*A, present in COTT may be integral to supplying nitrogen required for the biosynthesis of a variety of metabolic intermediates.^99,100^ COTT had the highest abundance of serine protease, subtilisin family, and it is predicted that these exoproteases break down proteins present in the environment in response to low levels of available nitrogen.^101^Additional file 2: Supplementary Table S24 contains this gene inventory.

### 3.13. Heavy metal resistance

Crude oil and heavy metal pollution occur simultaneously in soil.^102^ Toxic concentrations of cobalt (Co), nickel (Ni) and zinc (Zn) are detoxified by cation efflux mechanisms in bacteria.^103,104^ The COTT genome contains genes associated with cation antiporter cobalt–zinc–cadmium CzcCBA. Czc in *C*. *metallidurans* allowed its survival in metal-rich environments.^105^ Zinc and cadmium transporter, ZipB, is a ZIP zinc transporter protein, which controls the influx of zinc into the cytoplasm from outside of the cell and transport of cadmium^106,107^ and it was identified in COTT.

One means of relieving copper excess is through Cu^2+^ export facilitated by different systems, e.g. Cus, Cop and Cut, which contribute to copper homeostasis (Cu^2+^ import, export, detoxification) in bacteria.^108,109^ Importantly, high levels of Cu^2+^ disrupt Fe–S clusters which in turn affects disulphide bond formation and correct protein folding.^110^ The multi-protein complex that controls copper efflux, CusCBA, was detected in COTT. Copper resistance phosphate regulon response regulator (CusR) was also detected in COTT, which under anaerobic conditions, activates transcription of the *cus* operon.^109^ COTT also possesses genes in the copper inducible *cop* operon which aids in regulating copper resistance and detoxification, as well as heavy metal tolerance.^111^Additional file 2: Supplementary Table S24 contains this data.

### 3.14. Oxidative stress

Environmental pollutants can serve as oxidants that undergo biotransformation to produce free radical oxides, cations and anions.^112^ The COTT genome has three genes that code for Cu/Zn superoxide dismutase SOD2, and one Fe/Mn superoxide dismutase SOD1. After SOD converts radical species to H_2_O_2_, catalase neutralizes H_2_O_2_ into harmless H_2_O and O_2_.^113^ An alkyl hydroperoxide reductase (*ahp*C) encoded gene was found in COTT genome; it is known to be directly involved in H_2_O_2_ detoxification and it also plays an important role in biofilm formation.^114^ The COTT genome had three diheme cytochrome *c* peroxidase genes which are responsible for H_2_O_2_ reduction.^115^ The presence of oxidative stress proteins may enable COTT to cope with shifts in sediment oxygen concentration.^116^

### 3.15. Antibiotic resistome of COTT

There has been an ongoing, dedicated search for new antibiotics for the treatment of multidrug-resistant (MDR) infections caused by bacteria. Based on antiSMASH analysis, there were genes encoding novel antimicrobial compounds in the COTT genome (Additional file 2: Supplementary Table S26). Flexirubin and carotenoid clusters were identified in antiSMASH (Additional file 1: Supplementary Fig. S2) and are likely expressed to produce pigments to give colonies of this genus (*chryseos* = golden) a yellow to orange colour.^117^

The COTT strain was resistant to aztreonam, kanamycin, chloramphenicol, ampicillin and erythromycin but was susceptible to imipenem-EDTA, streptomycin, ciprofloxacin and trimethoprim in *in vitro* disc assays (Additional file 1: Supplementary Fig. S3 and Additional file 2: Supplementary Table S27). The resistome of COTT consisted of 21 β-lactam resistance genes, 13 cationic antimicrobial peptide (CAMP) resistance genes, 5 vancomycin resistance genes and 2 tetracycline resistance genes (Additional file 2: Supplementary Table S28). The COTT genome was the only *C*. *oranimense* genome containing the bla regulator protein BlaR1 (K02172), which largely controls β-lactam antibiotic resistance^118^ and membrane carboxypeptidase (penicillin-binding protein) which would render COTT resistant to penicillin (Additional file 2: Supplementary Table S18 and S28). There were also three phenicol and two bacteriocin genes identified in COTT (Additional file 2: Table S28).

The COTT genome has 16 gene copies encoding the major facilitator superfamily (MFS) of permeases (Additional file 2: Table S18). Notably, among these were the DHA1 and DHA2 family of multidrug resistance proteins which confer resistance to tetracycline, bicyclomycin/chloramphenicol; the FSR family of transporters which enables resistance to fosmidomycin, EmrB/QacA subfamily drug resistance transporter and PAT family β-lactamase induction signal transducer, AmpG, which allow ampicillin resistance (Additional file 2: Supplementary Tables S7 and S9). A summary of the AMR and multi-drug genes annotated in this genome and corresponding mechanisms are provided in Table 4. In this context, this report may have additional clinical value.

**Table 4.** Antimicrobial and multi-drug resistance genes

^1^Antimicrobial resistance mechanism	Genes
Antibiotic activation enzyme	*Kat*G
Antibiotic inactivation enzyme	CIA family, IND family
Antibiotic target in susceptible species	*Ddl, EF-G, EF-Tu, folA, Dfr, fol*P*, gyr*A*, gyr*B*, inh*A*, fab*I*, Iso-tRNA, kas*A*, Mur*A*, rho, rpo*B*, rpo*C*, S10p, S12p*
Gene conferring resistance via absence	*gid*B
Protein altering cell wall charge conferring antibiotic resistance	G*dp*D
Regulator modulating expression of antibiotic resistance genes	O*xy*R

Multi-drug resistance mechanism	
Virginiamycin A acetyltransferase	*vat*
Fluoroquinolone resistance protein	*qnr, mcb*G
Outer membrane protein, multidrug efflux system	*opr*M*, emh*C*, ttg*C*, cus*C*, ade*K*, sme*F*, mtr*E*, cme*C*, ges*C
P-type Cu+ transporter—platinum resistance	*cop*A*, ctp*A*, ATP7*
Serine beta-lactamase-like protein LACTB, mitochondrial	*LACTB*
Beta-lactamase class A	*pen*P
Chloramphenicol O-acetyltransferase type A	*cat*A
ATP-binding cassette, subfamily B, multi-drug efflux pump	*man*C*, cps*B
ATP-binding cassette, subfamily B, multi-drug efflux pump	*rfb*A*, rml*A*, rff*H

^1^The k-mer-based AMR genes detection method was used, which utilizes PATRIC’s curated collection of representative AMR gene sequence variants and assigns to each AMR gene functional annotation, broad mechanism of antibiotic resistance, drug class and, in some cases, a specific antibiotic it confers resistance to. Please note, that the presence of AMR-related genes (even full length) in a given genome does not directly imply an antibiotic-resistant phenotype. It is important to consider specific AMR mechanisms and especially the absence/presence of SNP mutations conveying resistance. MDR genes were mined from the KEGG database.

This work predicted distinctive metabolic capabilities that are unique to COTT (Fig. 5) and analysis of the COTT resistome indicated that these findings have clinical value. Notably, we also provided gene-based evidence that COTT is capable of producing several biocatalysts that may be important to different industrial processes.

## Supplementary Material

dsad025_suppl_Supplementary_File_S1

dsad025_suppl_Supplementary_File_S2

dsad025_suppl_Supplementary_File_S3

dsad025_suppl_Supplementary_File_S4

## Data Availability

The whole genome sequence of *C*. *oranimense* TT (COTT) is available in DDBJ/ENA/GenBank repository [JAPDSL000000000, https://www.ncbi.nlm.nih.gov/search/all/?term=JAPDSL000000000], Genomes OnLine Database (GOLD) [Project ID: Gp0615513, https://gold.jgi.doe.gov/projects?id=Gp0615513] and JGI IMG/M database [https://img.jgi.doe.gov/cgi-bin/m/main.cgi?section=TaxonDetail&page=taxonDetail&taxon_oid=2945865373].
