# Supplementary material for: Genome features of a novel hydrocarbonoclastic Chryseobacterium oranimense strain and its comparison to bacterial oil-degraders and to other C. oranimense strains
Source: DNA Res. 2023 Nov 11;30(6):dsad025. doi: 10.1093/dnares/dsad025 (PMC10710014; doi:10.1093/dnares/dsad025)
Supplement: dsad025_suppl_Supplementary_File_S1 [file dsad025_suppl_supplementary_file_s1.pptx]

## Slide 1
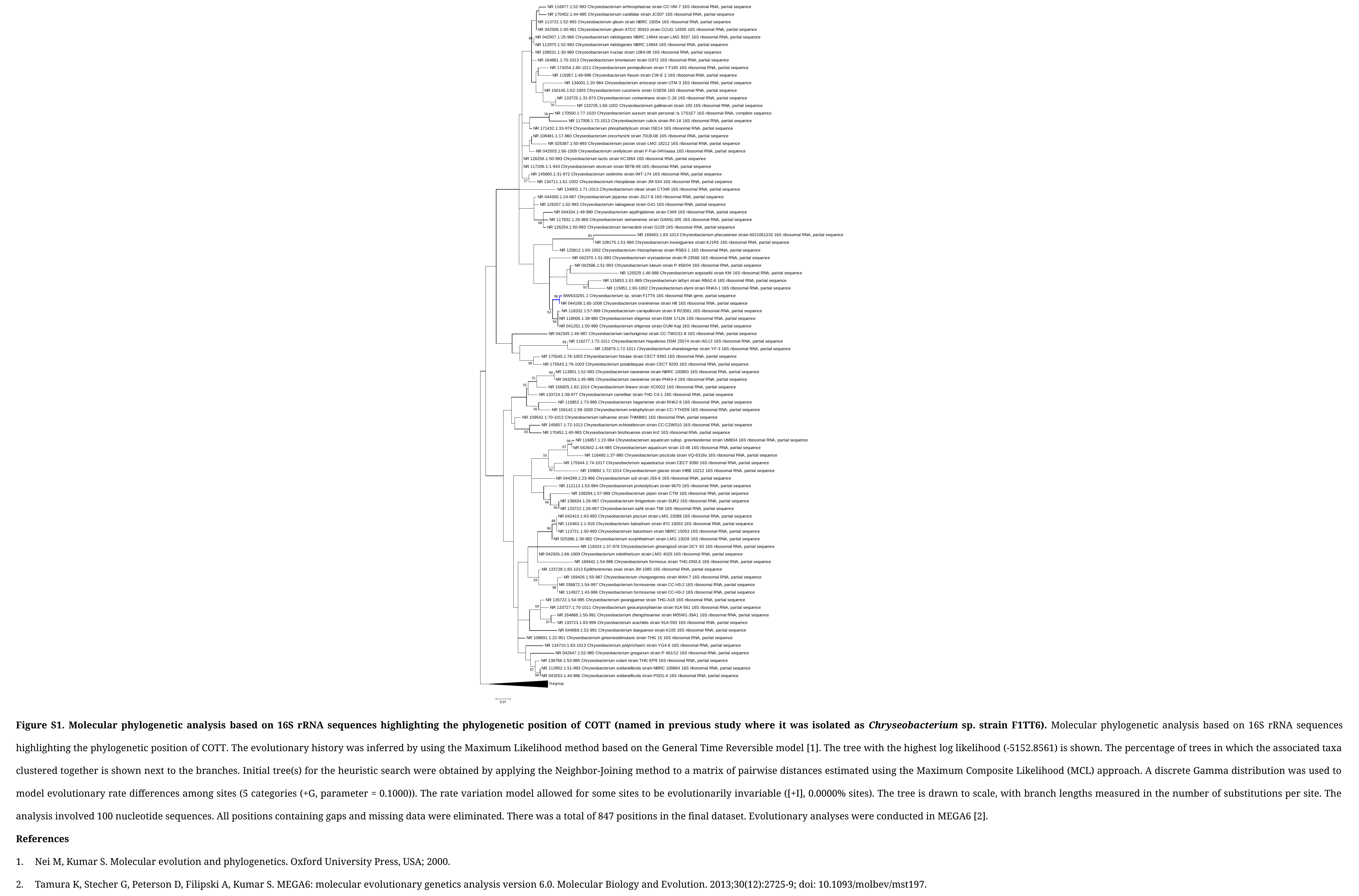

Figure S1. Molecular phylogenetic analysis based on 16S rRNA sequences highlighting the phylogenetic position of COTT (named in previous study where it was isolated as Chryseobacterium sp. strain F1TT6). Molecular phylogenetic analysis based on 16S rRNA sequences highlighting the phylogenetic position of COTT. The evolutionary history was inferred by using the Maximum Likelihood method based on the General Time Reversible model [1]. The tree with the highest log likelihood (-5152.8561) is shown. The percentage of trees in which the associated taxa clustered together is shown next to the branches. Initial tree(s) for the heuristic search were obtained by applying the Neighbor-Joining method to a matrix of pairwise distances estimated using the Maximum Composite Likelihood (MCL) approach. A discrete Gamma distribution was used to model evolutionary rate differences among sites (5 categories (+G, parameter = 0.1000)). The rate variation model allowed for some sites to be evolutionarily invariable ([+I], 0.0000% sites). The tree is drawn to scale, with branch lengths measured in the number of substitutions per site. The analysis involved 100 nucleotide sequences. All positions containing gaps and missing data were eliminated. There was a total of 847 positions in the final dataset. Evolutionary analyses were conducted in MEGA6 [2].
References
1.	Nei M, Kumar S. Molecular evolution and phylogenetics. Oxford University Press, USA; 2000.
2.	Tamura K, Stecher G, Peterson D, Filipski A, Kumar S. MEGA6: molecular evolutionary genetics analysis version 6.0. Molecular Biology and Evolution. 2013;30(12):2725-9; doi: 10.1093/molbev/mst197.

## Slide 2
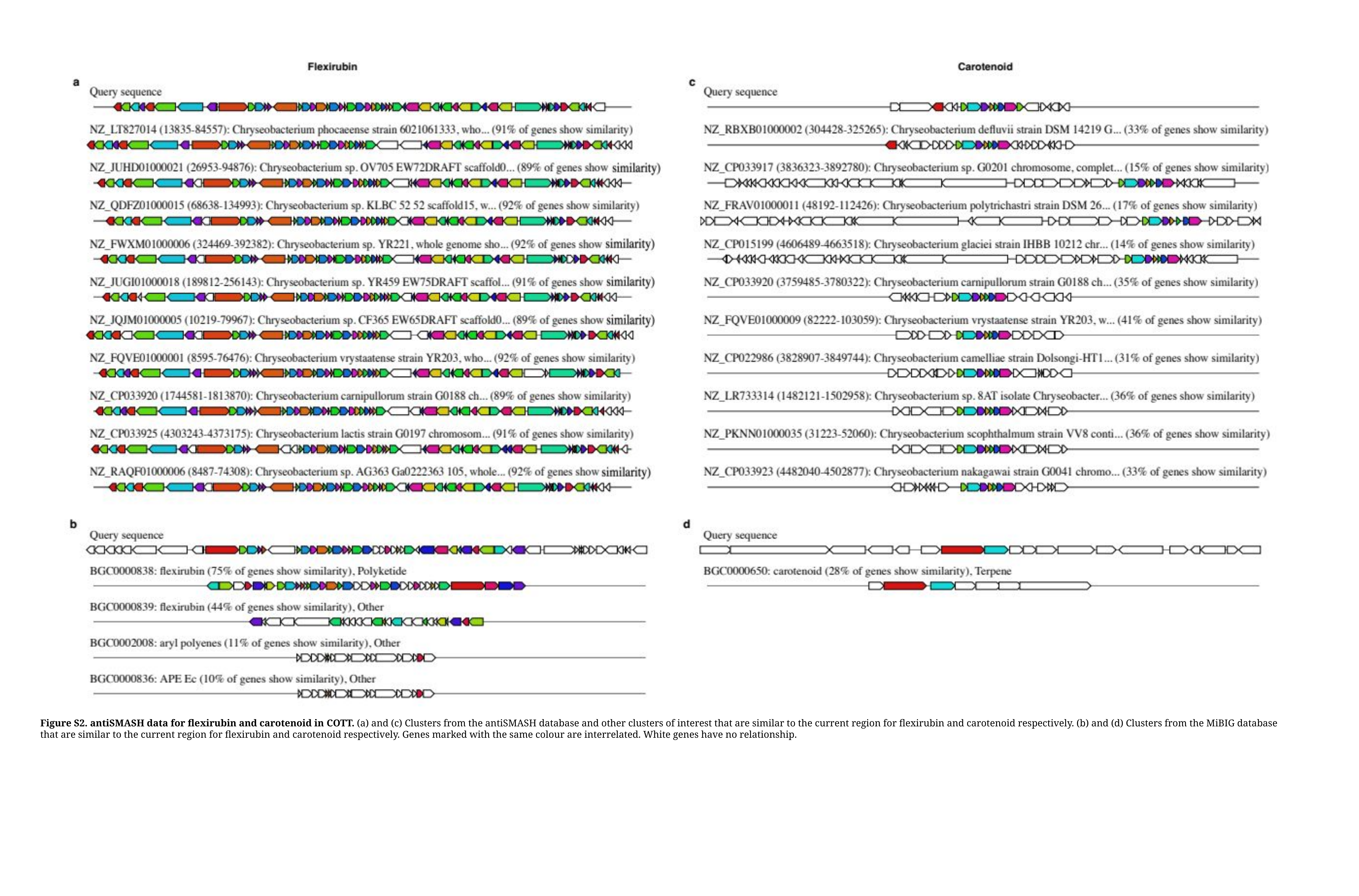

Figure S2. antiSMASH data for flexirubin and carotenoid in COTT. (a) and (c) Clusters from the antiSMASH database and other clusters of interest that are similar to the current region for flexirubin and carotenoid respectively. (b) and (d) Clusters from the MiBIG database that are similar to the current region for flexirubin and carotenoid respectively. Genes marked with the same colour are interrelated. White genes have no relationship.

## Slide 3
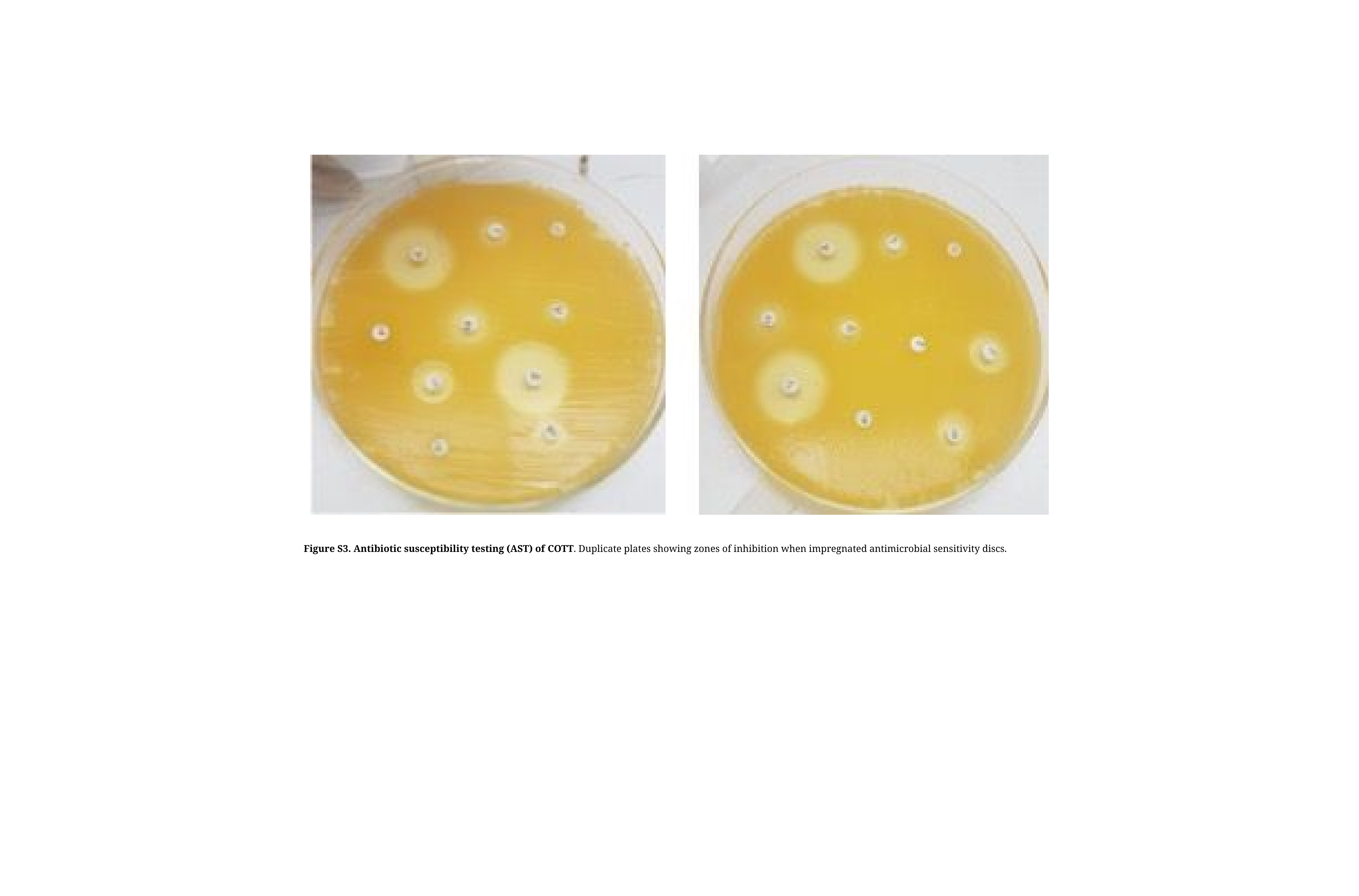

Figure S3. Antibiotic susceptibility testing (AST) of COTT. Duplicate plates showing zones of inhibition when impregnated antimicrobial sensitivity discs.
