# Supplementary material for: Genome features of a novel hydrocarbonoclastic Chryseobacterium oranimense strain and its comparison to bacterial oil-degraders and to other C. oranimense strains
Source: DNA Res. 2023 Nov 11;30(6):dsad025. doi: 10.1093/dnares/dsad025 (PMC10710014; doi:10.1093/dnares/dsad025)
Supplement: dsad025_suppl_Supplementary_File_S3 [file dsad025_suppl_supplementary_file_s3.docx]

**Additional File : Supplementary Notes and Analysis**

**Note and analysis S1: Genome project history**

***Sequencing data quality control***

Sequencing quality distribution: Sequencing quality distribution was examined over the full length of all sequences, to detect any sites (base positions) with an unusually low sequencing quality, where incorrect bases may be incorporated at abnormally high levels. For detailed sequencing quality distribution, please refer to Figure N1.

**a** **b**


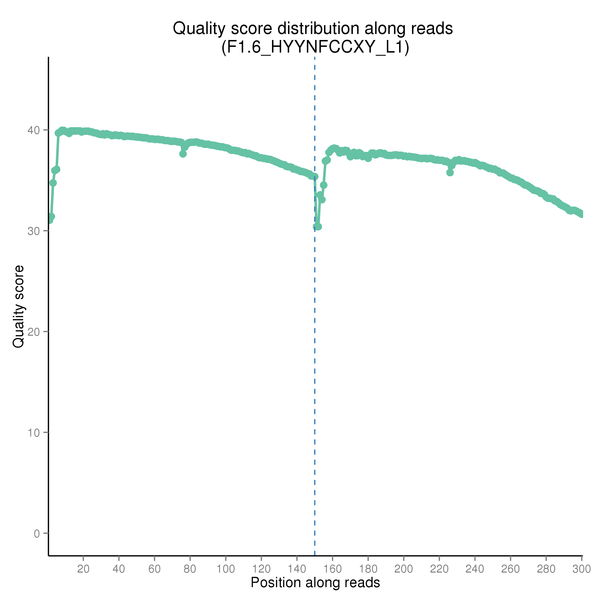

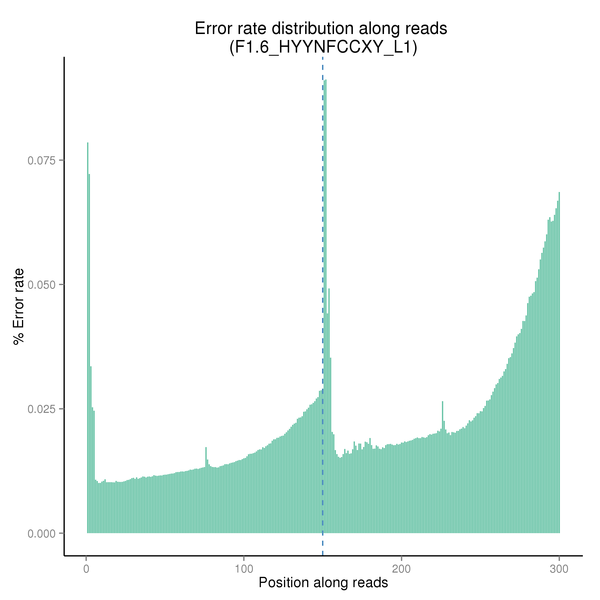


**Figure N1. Sequencing quality distribution.** (**a**) Distribution of sequencing quality. The x-axis shows the base position within a sequencing read, and the y-axis shows the average Phred score of all reads at each position. (Pair-end sequencing data are plotted together, with the first PE150 bp representing read 1 and the following PE150 bp for read 2.); (**b**) distribution of sequencing errors. The x-axis shows the base position within a sequencing read, and the y-axis shows the average error rate of all reads at each position. (Pair-end sequencing data are plotted together, with the first PE150 bp representing read 1 and the following PE150 bp for read 2.).

Distribution of sequencing errors: Sequencing error rate distribution was examined over the full length of all sequences, to detect any sites (base positions) with an unusually high error rate, where incorrect bases may be incorporated at abnormally high levels. For detailed sequencing error distribution, please refer to Figure N1.

Sequencing data filtration: Raw data obtained from sequencing contains adapter contamination and low-quality reads. These sequencing artifacts may increase the complexity of further analyses, so quality control steps to remove them were utilized. Consequently, all the further analyses are based on the clean reads. Please refer to Figure N2.


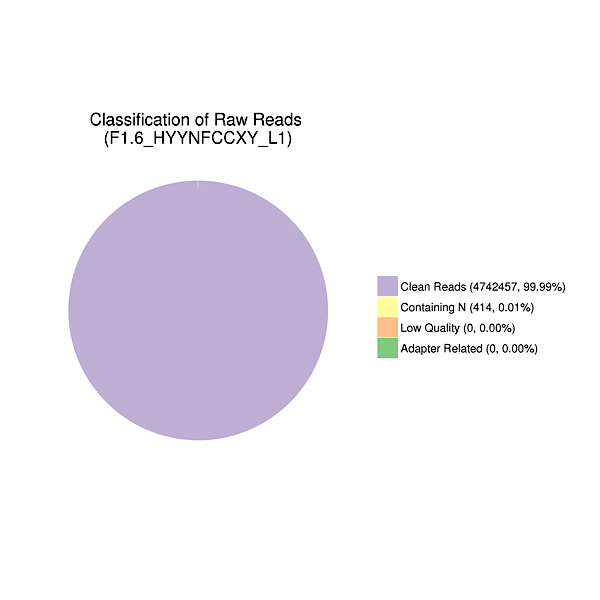


**Figure N2. Classification of the sequenced reads.** (1) Adapter related: The proportion of filtered reads containing adapters in total reads. (2) Containing N: The proportion of filtered reads containing more than 10% Ns in total reads.(3) Low quality: The proportion of filtered reads for low quality in total reads. (4) Clean reads: The proportion of clean reads in raw reads.

Statistic of sequencing data: Totally 1.4 G raw data were sequenced from this run, with 1.4 G clean data generated after filtering low-quality data, indicating the sufficient amount of data production. Currently for Illumina sequencers, the per base error rate is generally lower than 1%, and the highest acceptable threshold is 6%. Statistics of sequencing data are listed in Table N1.

Table N1 Statistics of sequencing data

| **Raw reads** | **Raw data (G)** | **Clean data (G)** | **Effective (%)** | **Error (%)** | **Q20 (%)** | **Q30 (%)** | **GC (%)** |
| --- | --- | --- | --- | --- | --- | --- | --- |
| 4742871 | 1.4 | 1.4 | 99.99 | 0.03 | 94.28 | 87.52 | 37.95 |

The details for the sequencing data statistics are as follows:

(1) Sample name: Sample name.

(2) Lane: The flowcell ID and lane number during the sequencing (FlowcellID_LaneNumber).

(3) Raw reads: The number of sequencing reads pairs; four lines will be considered as one unit according to FASTQ format.

(4) Raw data (G): The original sequence data volume.

(5) Clean data (G): The sequence data volume calculated by clean data..

(6) Effective (%): The ratio of clean data to raw data.

(7) Error (%): Overall error rate of base.

(8) Q20 (%): The percentage of bases with higher Phred score than 20.

(9) Q30 (%): The percentage of bases with higher Phred score than 30.

(10) GC: The percentage of G and C in the total bases.

***BUSCO data***

The BUSCO score was 99.7%; which is considered to be within acceptable limits to assess species coverage and assembly quality.

***Mapping statistics***

For the current 4,457,049 bp reference genome, the mapping rate of *C. oranimense* TT is 90.79%. Referring to the reference genome (without Ns), the average depth is 296.89X, and more than 1X coverage reaches 88.30%. This result is in the qualified normal range and thus was served in the subsequent variation detection and related analyses. The mapping rate and coverage results for the reference genome are listed in Table N2.

Table N2 Statistics of reference genome (GCA_000612925.1)

| **Total length** | **GC content (%)** | **Gap rate (%)** | **N50 length** | **N90 length** |
| --- | --- | --- | --- | --- |
| 4,457,049 | 37.68 | 0.00 | 689,374 | 234,272 |

The detailed information of reference are as follows:

(1) Seq number: the total number of the assembled genomic sequences.

(2) Total length: the total length of the assembled genomic sequences.

(3) GC content: the GC content of the reference genome.

(4) Gap rate: the proportion of unknown sequence (N) in the reference genome assembly.

(5) N50 length: the length of scaffold N50, of which 50% of the sequences is higher than this level.

(6) N90 length: the length of scaffold N90, of which 90% of the sequences is higher than this level.

The mapping rate of *C. oranimense* TT reflecting the similarity between *C. oranimense* TT and the reference genomes is listed in Table N3. The depth and coverage are indicators of the evenness and homology with the reference genome.

Table N3 Statistics of mapping rate, depth, and coverage

| **Mapped reads** | **Total reads** | **Mapping rate (%)** | **Average depth (x)** | **Coverage at least 1X (%)** | **Coverage at least 4X (%)** |
| --- | --- | --- | --- | --- | --- |
| 8,611,670 | 9,484,914 | 90.79 | 296.89 | 88.30 | 88.26 |

The details for mapping statistics are as follows:

(1) Sample: Sample names.

(2) Mapped reads: The number of clean reads mapped to the reference, including both single-end reads and reads in pairs.

(3) Total reads: Total number of effective reads in clean data.

(4) Mapping rate: The ratio of Mapped reads to the total sequenced clean reads.

(5) Average depth: The average depth of mapped reads at each site, calculated by the total number of bases in the mapped reads dividing by size of the assembled genome.

(6) Coverage at least 1X: The percentage of the assembled genome with more than one read at each site.

(7) Coverage at least 4X: The percentage of the assembled genome with ≥4X coverage at each site.

***SNP detection and annotation***

ANNOVAR was used to perform annotation of detected SNPs. The details for SNP detection and annotation statistics are provided in Table N4. The credibility of detected SNPs, we checked the distribution of support reads number, SNP quality, as well as the distance between adjacent SNPs. The results are shown in Figure N3. Accordingly, the whole-genome SNP mutations could be classified into six categories. The frequency of each type is shown in Figure N4.

Table N4 Statistics of SNP detection and annotation

| **Upstream** | **Exonic** | | | | **Intronic** | **Splicing** | **Downstream** | **Upstream/Downstream** | **Intergenic** | **ts** | **tv** | **ts/tv** | **Het rate(‰)** | **Total** |
| --- | --- | --- | --- | --- | --- | --- | --- | --- | --- | --- | --- | --- | --- | --- |
|  | **Stop gain** | **Stop loss** | **Synonymous** | **Non-synonymous** |  |  |  |  |  |  |  |  |  |  |
| 891 | 39 | 16 | 39894 | 9072 | 0 | 0 | 864 | 5010 | 0 | 46368 | 16174 | 2.867 | 0.095 | 62542 |

The details for SNP detection and annotation statistics are as follows:

(1) Sample: Sample name;

(2) Upstream: SNPs located within 1 kb upstream (away from transcription start site) of the gene.

(3) Exonic: SNPs located in exonic region; Non-synonymous: single nucleotide mutation with changing amino acid sequence; Stop gain/loss: a nonsynonymous SNP that leads to the introduction/removal of stop codon at the variant site; Synonymous: single nucleotide mutation without changing amino acid sequence;

(4) Intronic: SNPs located in intronic region;

(5) Splicing: SNPs located in the splicing site (2 bp range of the intron/exon boundary).

(6) Downstream: SNPs located within 1 kb downstream (away from transcription termination site) of the gene region.

(7) Upstream/Downstream: SNPs located within the < 2 kb intergenic region, which is in 1 kb downstream or upstream of the genes.

(8) Intergenic: SNPs located within the > 2 kb intergenic region.

(9) ts: Transitions, a point mutation that changes a purine nucleotide to another purine (A <-> G) or a pyrimidine nucleotide to another pyrimidine (C <-> T). Approximately two out of three SNPs are transitions.

(10) tv: Transversions, the substitution of a (two ring) purine for a (one ring) pyrimidine or vice versa.

(11) ts/tv: The ratio of transitions to transversions.

(12) Het rate: Genome-wide heterozygous rate, calculated by the ratio of heterozygous SNPs to the total number of genome bases.

(13) Total: The total number of SNPs.


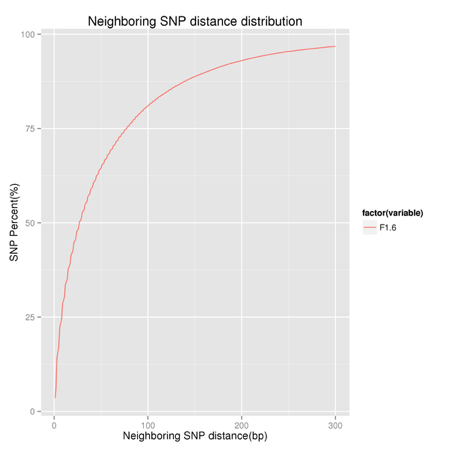

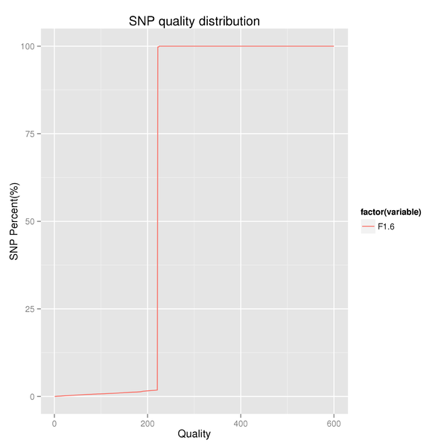

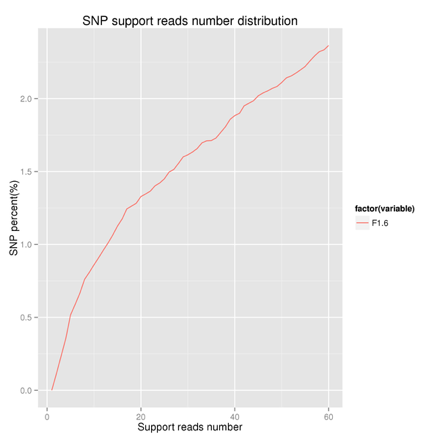


**Figure N3. Cumulative distribution of SNP quality.** Note: These figures show the quality distribution of SNPs, from top to bottom, the distribution of SNP support reads number, the distribution of distances between adjacent SNPs and the cumulative distribution of SNP quality.


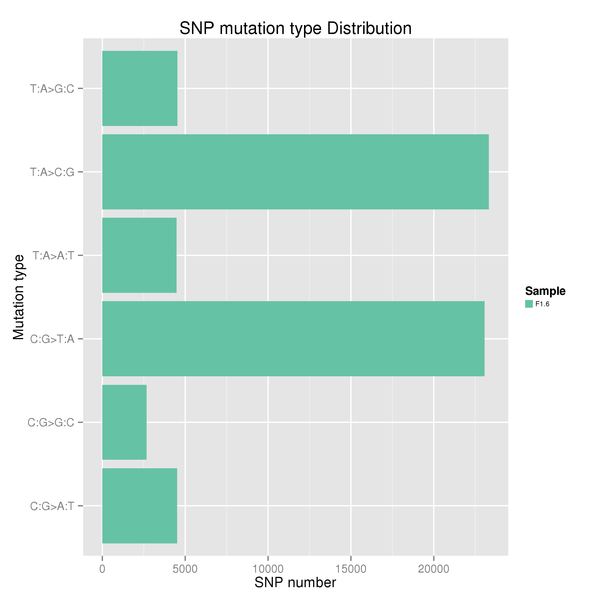


**Figure N4. Frequency of SNP mutations.** The x-axis represents the number of the SNPs and y-axis indicates the mutation types.

***InDel detection and annotation***

InDel annotation statistics are stated in Table N5. Length distribution of CDS-located InDels can be viewed in Figure N5.

Table N5 Statistics of InDel detection and annotation

| **Upstream** | **Exonic** | | | | | | **Intronic** | **Splicing** | **Downstream** | **Upstream/Downstream** | **Intergenic** | **Insertion** | **Deletion** | **Het rate(‰)** | **Total** |
| --- | --- | --- | --- | --- | --- | --- | --- | --- | --- | --- | --- | --- | --- | --- | --- |
|  | **Stop gain** | **Stop loss** | **Frameshift deletion** | **Frameshift insertion** | **Non-frameshift deletion** | **Non-frameshift insertion** |  |  |  |  |  |  |  |  |  |
| 18 | 1 | 2 | 14 | 22 | 4 | 2 | 0 | 0 | 65 | 238 | 0 | 219 | 170 | 0.000 | 389 |

The details of InDel annotation statistics are as follows:

(1) Sample: Sample names.

(2) Upstream: InDels located within 1 kb upstream (away from transcription start site) of the gene.

(3) Exonic: InDels located in exonic region; Stop gain/loss: InDel that leads to the introduction/removal of stop codon at the variant site; Frameshift deletion/insertion: InDel mutation changing the open reading frame with deletion or insertion; Non-Frameshift deletion/insertion: InDel mutation without changing the open reading frame with deletion or insertion sequences of 3 or multiple of 3 bases;

(4) Intronic: InDel located in intronic region;

(5) Splicing: InDel located in the splicing site (2 bp range of the intron/exon boundary).

(6) Downstream: InDel located within 1 kb downstream (away from transcription termination site) of the gene region.

(7) Upstream/Downstream: InDel located within the < 2 kb intergenic region, which is in 1 kb downstream or upstream of the genes.

(8) Intergenic: InDel located within the > 2 kb intergenic region.

(9) Het rate: InDel heterozygous rate, calculated by the ratio of InDels to the total number of genome bases.

(10) Total: The total number of InDels.


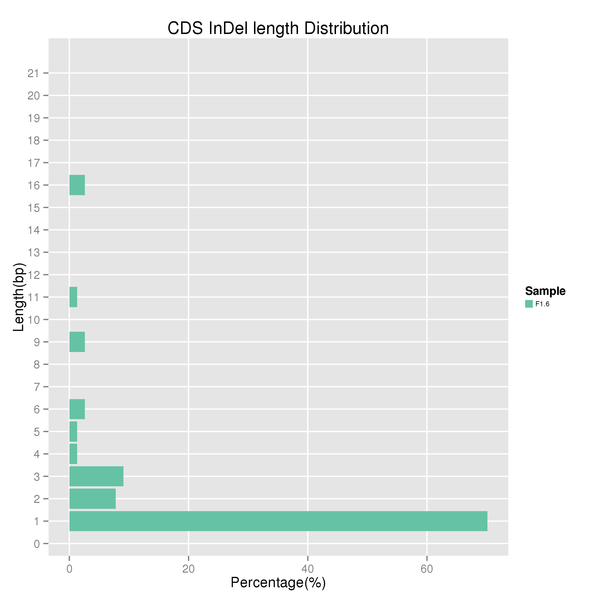


**Figure N5. Length distribution of CDS-located InDels.** The x-axis represents the proportion of the InDels with a certain length, and y-axis indicates the length of the InDels.

***SV detection and annotation***

SVs detection statistics are shown in Table N6. The length distribution of the SVs can be found in Figure N6.

Table N6 Statistics of SV detection and annotation

| **Upstream** | **Exonic** | **Downstream** | **Intronic** | **Upstream/Downstream** | **Intergenic** | **Splicing** | **INS** | **DEL** | **INV** | **ITX** | **CTX** | **Total** |
| --- | --- | --- | --- | --- | --- | --- | --- | --- | --- | --- | --- | --- |
| 1 | 58 | 0 | 0 | 4 | 0 | 0 | 1 | 54 | 9 | 162 | 30 | 256 |

The details of SV detection statistics are as follows:

(1) Sample: Sample names.

(2) Upstream: SVs located within 1 kb upstream (away from transcription start site) of the gene.

(3) Exonic: SVs located in exonic region.

(4) Intronic: SVs located in intronic region.

(5) Downstream: SVs located within 1 kb downstream (away from transcription termination site) of the gene region.

(6) Upstream/Downstream: SVs located within the < 2 kb intergenic region, which is in 1 kb downstream or upstream of the genes.

(7) Intergenic: SVs located within the > 2 kb intergenic region.

(8) Splicing: SVs located in the splicing site (2 bp range of the intron/exon boundary).

(9) INS: Insersion.

(10) DEL: Deletion.

(11) INV: Inversion.

(12) ITX: Intra-chromosomal translocations.

(13) CTX: Inter-chromosomal translocations.

(14) Total: The total number of SVs.


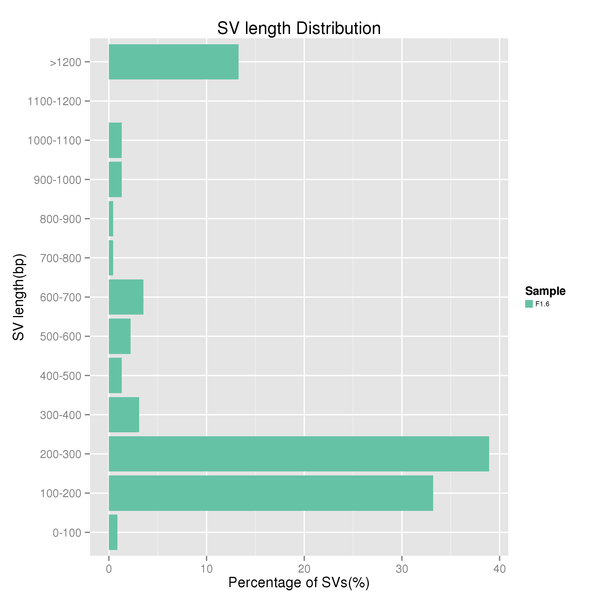


**Figure N6. Length distribution of SVs.** The x-axis represents the proportion of the SVs with certain range of length, and the y-axis indicates the certain length range of the SVs. Note, the length of DNA insert in library construction impacts the SVs detection greatly.

***CNV detection and annotation***

Structural variation showing deletions or duplications in the genome are shown in Table N7. The distribution of CNVs on the genome SVs can be found in Figure N7.

Table N7 Statistics of CNV detection and annotation

| **Upstream** | **Exonic** | **Intronic** | **Downstream** | **Upstream/Downstream** | **Intergenic** | **Duplication** | **Deletion** | **Duplication length (bp)** | **Deletion length (bp)** | **Total** |
| --- | --- | --- | --- | --- | --- | --- | --- | --- | --- | --- |
| 1 | 125 | 0 | 0 | 3 | 0 | 12 | 120 | 37900 | 523900 | 132 |

The details of CNV detection and annotation are as follows:

(1) Sample: Sample names.

(2) Upstream: CNVs located within 1 kb upstream (away from transcription start site) of the gene.

(3) Exonic: CNVs located in exonic region.

(4) Intronic: CNVs located in intronic region.

(5) Downstream: CNVs located within 1 kb downstream (away from transcription termination site) of the gene region.

(6) Upstream/Downstream: CNVs located within the < 2 kb intergenic region, which is in 1 kb downstream or upstream of the genes.

(7) Intergenic: CNVs located within the > 2 kb intergenic region.

(8) Duplication: CNVs with increased copy number.

(9) Deletion: CNVs with decreased copy number.

(10) Duplication length (bp): The total length of CNV duplication.

(11) Deletion length (bp): The total length of CNV deletion.

(12) Total: The total number of CNVs.


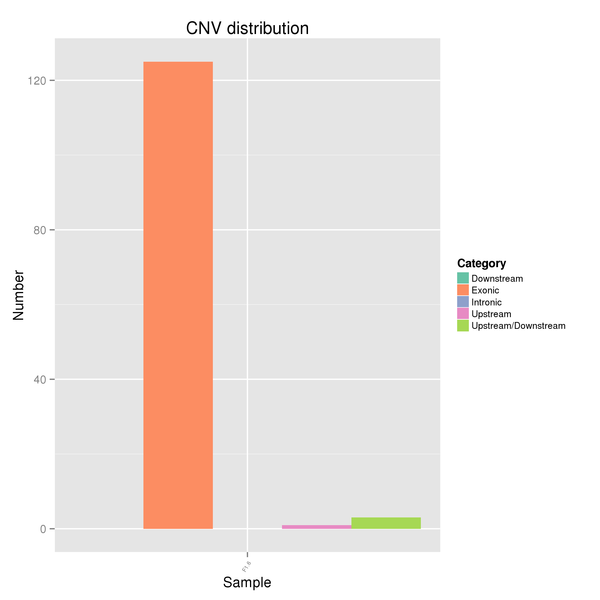


**Figure N7. CNV annotation.** The distribution of CNVs on the genome. The x-axis represents samples and the y-axis indicates the number of CNVs in different region.


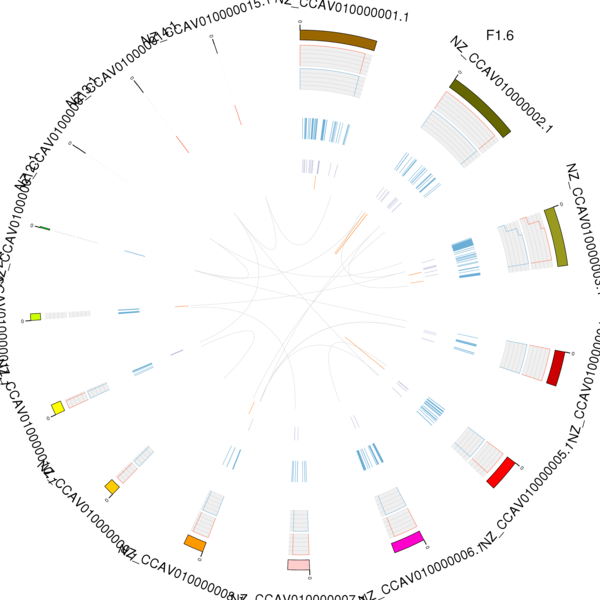


**Figure N8. Whole genome variations distribution.** From outer to inner: chromosome, SNP, InDel, CNV duplication, CNV deletion, SV insertion, SV deletion, SV inversion, SV ITX, SV CTX.

**Note and analysis S2 : BV-BRC genome data**

***Genome assembly and annotation data***

The assembled genome was submitted to the Comprehensive Genome Analysis service in BV-BRC. This assembled genome had 12 contigs, with the total length of 4,265,626 bp and an average G+C content of 37.91% (Table N8).

Table N8 Assembly details

| **Assembly** | **Spades** |
| --- | --- |
| # contigs (>= 0 bp) | 12 |
| Total length (>= 0 bp) | 4,265,626 |
| GC (%) | 37.91 |
| N50 | 3,153,741 |
| N75 | 864759 |
| L50 | 1 |
| L75 | 2 |
| # N's per 100 kbp | 0 |

This genome has 4,043 protein coding sequences (CDS), 66 transfer RNA (tRNA) genes, and 4 ribosomal RNA (rRNA) genes. The annotated features are summarized in Table N9.

Table N9 Annotated genome features - BV-BRC

| CDS | 4,043 |
| --- | --- |
| tRNA | 66 |
| rRNA | 4 |
| Partial CDS | 0 |
| Miscellaneous RNA | 0 |
| Repeat Regions | 0 |

The annotation included 1,777 hypothetical proteins and 2,266 proteins with functional assignments (Table N10). The proteins with functional assignments included 804 proteins with Enzyme Commission (EC) numbers, 701 with Gene Ontology (GO) assignments, and 605 proteins that were mapped to KEGG pathways. BV-BRC annotation includes two types of protein families, and this genome has 3,776 proteins that belong to the genus-specific protein families (PLFams), and 3,839 proteins that belong to the cross-genus protein families (PGFams).

Table N10 Protein features - BV-BRC

|  | **Number** | |
| --- | --- | --- |
| Genomic features |  | |
| DNA, total number of bases | 4,265,626 | |
| GC content | 37.91 | |
| CDS | 4043 | |
| tRNA | 66 | |
| rRNA | 4 | |
| Protein features |  | |
| Hypothetical proteins | [1777](https://patricbrc.org/view/Genome/421058.14#view_tab=features&filter=and(eq(annotation,PATRIC),eq(product,hypothetical+protein),eq(feature_type,CDS))) | |
| Proteins with functional assignments | [2266](https://patricbrc.org/view/Genome/421058.14#view_tab=features&filter=and(eq(annotation,PATRIC),ne(product,hypothetical+protein),eq(feature_type,CDS))) | |
| Proteins with EC number assignments | 804 | |
| Proteins with GO assignments | [701](https://patricbrc.org/view/Genome/421058.14#view_tab=features&filter=and(eq(annotation,PATRIC),eq(go,*))) | |
| Proteins with Pathway assignments | 605 | |
| Proteins with PATRIC genus-specific family (PLfam) assignments | [3776](https://patricbrc.org/view/Genome/421058.14#view_tab=features&filter=and(eq(annotation,PATRIC),eq(plfam_id,PLF*))) | |
| Proteins with PATRIC cross-genus family (PGfam) assignments | [3839](https://patricbrc.org/view/Genome/421058.14#view_tab=features&filter=and(eq(annotation,PATRIC),eq(pgfam_id,PGF*))) | |
| Specialty Genes | Source | Genes |
| Transporter | TCDB | 2 |
| Antibiotic Resistance | PATRIC | 28 |
| Antibiotic Resistance | CARD | 1 |
| Antibiotic Resistance | NDARO | 1 |

***BV_BRC – Initial annotation data retrieval***

The genome was annotated using RASTtk and assigned a unique genome identifier of 421058.16. This genome is in the superkingdom Bacteria and was annotated using genetic code 11. The taxonomy of this genome is: cellular organisms > Bacteria > FCB group > *Bacteroidetes/Chlorobi* group > *Bacteroidetes* > *Flavobacteriia* > *Flavobacteriales* > *Weeksellaceae* > *Chryseobacterium* group > *Chryseobacterium* > *Chryseobacterium oranimense*. A circular graphical display of the distribution of the genome annotations is provided (Figure N9). This includes, from outer to inner rings, the contigs, CDS on the forward strand, CDS on the reverse strand, RNA genes, CDS with homology to known antimicrobial resistance genes, CDS with homology to know virulence factors, GC content and GC skew. The colours of the CDS on the forward and reverse strand indicate the subsystem that these genes belong to.


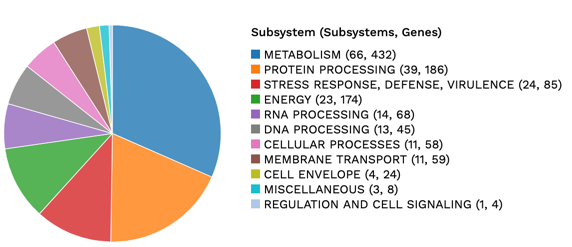

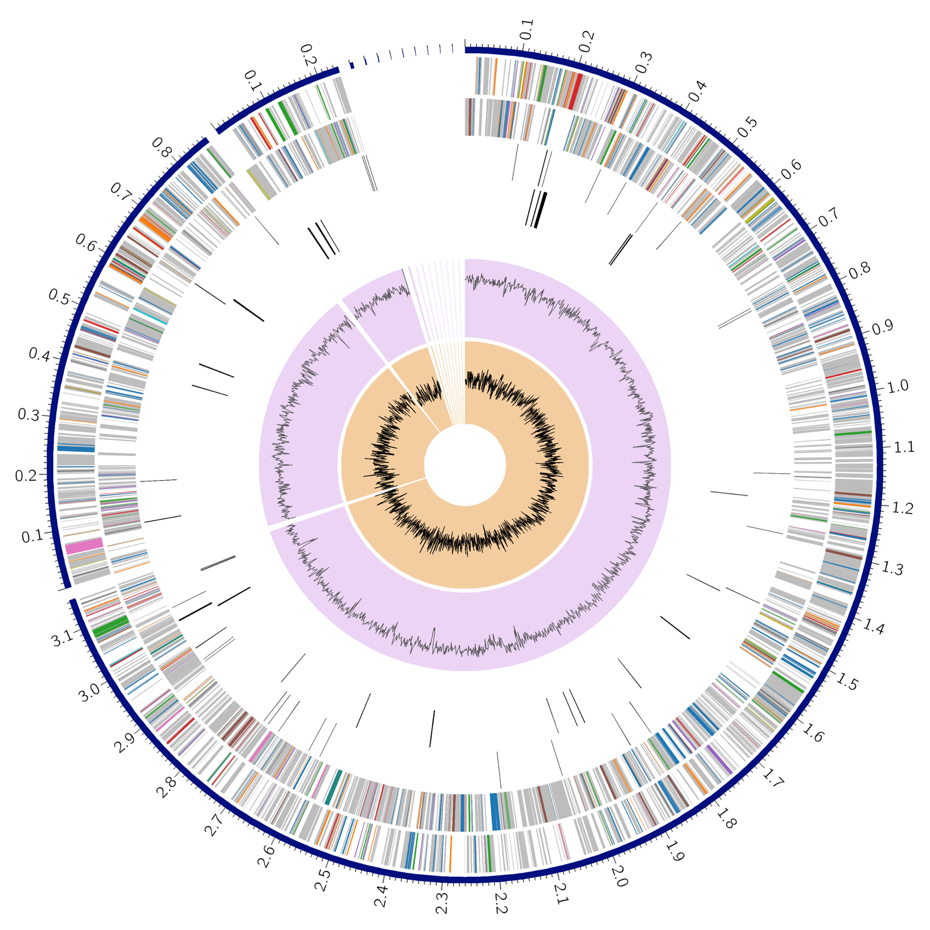


**Figure N9. Circular chromosome map of COTT.** The key represents subsystems in the genome.

**Note and analysis S3: *Genes only found in the COTT genome suggest a lifetime of adaptation***

*1. Global stress tolerance*

Members of the GNAT (general control non-repressible 5 (GCN5)-related N-acetyltransferases) superfamily are able to catalyze a diverse range of acetylation reactions [1] related to aminoglycoside antibiotic resistance, protein acetylation, including histone modification for gene activation, xenobiotic metabolism, and other cellular processes [2]. 14 genes encoding GNAT enzymes were found in the COTT genome.

*2. Cell wall/membrane/envelope biogenesis*

Extracellular stress that leads to disruption of periplasmic homeostasis of the bacterial envelope is managed by ESRs (envelope stress responses) [3]. ESRs active several regulatory signal cascades [4, 5]. ESRs are grouped into (i) RNA polymerase-associating alternative sigma factors, and (ii) two component signal transduction (2CST) systems [5]. The σE- and Cpx-regulated Cpx (conjugative pilus expression) ESRs are important to mitigating disruption of periplasmic function in Gram-negative bacteria that would have otherwise induced membrane permeabilization, hyperpolarization and eventual cell death [4, 6].

The COTT genome contains the following periplasmic protection systems: (1) positive regulator of σE activity, rpoE; *σ*E is a minor sigma factor, that reduces the effects of heat shock on membrane and periplasmic proteins; phosphoethanolamine transferase for periplasmic glucans (OPG) of the alkaline phosphatase family (OPG was only found in the COTT genome), (2) periplasmic protein refolding chaperone Spy/CpxP family; the periplasm impounds enzymes that would otherwise be toxic to the cell in the cytoplasmic space, e.g., RNAses and alkaline phosphatases; these chaperones facilitate specific protein folding and regulation of cell division, and have structural homology to Spy [7], and (3) lipoprotein-anchoring transpeptidase ErfK/SrfK functions in periplasmic protein repair. COTT also has the highest abundance of genes coding for outer membrane protein OmpA and related peptidoglycan-associated (lipo)proteins**.**

Porins in the outer membrane allow passive diffusion of small molecules below ∼600 Da [8, 9]. This means that periplasmic proteins are more exposed to exogenous environmental stressors than cytoplasmic proteins. Only the COTT genome had two phosphate-selective porin enzymes; phosphate-selective porins are a family of outer bacterial membrane proteins [10]. These are anion-specific porins whose binding site has a higher substrate affinity for phosphate compared to chloride ions. Porin O has a higher affinity for polyphosphates, while porin P has a higher affinity for orthophosphate [11]. Phosphate increases the aerobic and denitrifying rates of oil degradation and are, therefore, needed for promoting microbial growth [12].

*3 Signal transduction mechanisms*

TCSs are ubiquitous signaling units that mediate regulatory systems for bacterial acclimation to various environmental changes by coupling environmental signals to gene expression [13]. There seemed to be multicomponent interactions between different regulatory systems and signal integration which suggest a much more complex signalling network for COTT than for the other *C. oranimense* strains [14]. This diversity of signalling domain combinations in the COTT strain, where several domain architectures stand out among regulators, suggests potential cross-talk between different signalling systems [15] in response to environmental challenges [16].

In addition to the transcriptional regulators of the OmpR, NarL, and NtrC families, COTT had transcriptional regulators with other DNA-binding domains (e.g., LytTR, AraC, Spo0A), which is also found in 6% of all bacteria. The REC domain and its combinations with different enzymatic domains (e.g., GGDEF, CheB, and HisK) are found in ~14% of bacteria including COTT [17].

Many response regulators can be found in a variety of distinct proteins associated with specific signalling domains [14]. The COTT genome harbours regulators for certain components of the signal transduction circuits, such as adenylate cyclases, diguanylate cyclases and phosphodiesterases, and serine/threonine protein kinases and phosphatases.

The following regulators were unique to the COTT genome and were not detected in the other *C*. *oranimense* genomes: two-component response regulator, PleD family, consisting of two REC domains and a diguanylate cyclase (GGDEF) domain (COG3706). These two-domain response regulators include Spo0A-type, CheY-CheB, and CheY (Phosphoacceptor domains)-GGDEF (diguanylate cyclase). GGDEF activity produces bis-(3′→5′)-cyclic diguanosine monophosphate (c-di-GMP), a secondary messenger in bacteria, and a CheY-like phosphoacceptor (or receiver [REC]) domain.

COTT also had a CHASE3-encoding gene (cyclase/histidine kinase-associated sensor) which is found in the extracellular portion of receptor-like proteins involved in osmotic stress response. CBS domain-containing proteins in COTT was reported to sense cellular energy levels in other bacteria [15].

*4. Denitrification*

Only the COTT genome had two genes encoding nitrous oxide reductase accessory protein, NosL. NosL is an accessory protein of the *nos* (nitrous oxide reductase) gene cluster and functions as a periplasmic copper chaperone for nitrous oxide reductase [18]. This protein is located in the outer membrane of Gram-negative bacteria and plays a complex role in denitrification which includes copper trafficking [19]. The multicopper enzyme nitrous oxide reductase catalyzes the final step of bacterial denitrification which reduces the greenhouse gas, N_2_O, to uncritical N_2_. Denitrifying Gammaproteobacteria were abundant in oil-polluted sediments suggesting that they may play a key role in hydrocarbon degradation in the environment [20]. Importantly, certain types of aliphatic and aromatic hydrocarbons are weakly soluble and recalcitrant to degradation which leads to high accumulation of total nitrogen in the environment. Crude oil increased denitrification and N_2_O emission rates as well as gene abundances, and led to more competition among N-cycling microbes in polluted environments [21]. It is proposed that NosL in the COTT genome would give this strain the competitive advantage over non-denitrifying bacteria in crude oil-contaminated soil.

*5. Motility and flagella apparatus*

MotA and MotB are cytoplasmic membrane proteins that form the force-generating unit of the flagellar motor in many flagellated bacteria [22]. Phylogenetic analyses of these C-terminal domains of MotB revealed that members of the MotB family and members of peptidoglycan-interaction domains diverged from each other early in evolutionary history before the presence of divergent isoforms [23]. As such, it is likely that MotB, found only in the COTT genome, may function as a member of the peptidoglycan-interaction domain. The following were found in the COTT genome which suggests a specialized role in exerting alternative cellular functions such as the control of second messenger levels since *Chryseobacterium* sp. are non-motile and non-flagellated [24-27]: (1) Flp pilus assembly protein TadB: type II secretion system, and tight adherence (*tad*) export gene cluster is required for attachment to substrates as it regulates the assembly of adhesive pili, (2) competence protein ComGC: Type IV pili are found on the surface of many pathogenic bacteria and it is involved in various functions, including biofilm formation, (3) P pilus assembly protein, chaperone PapD: also involved in the assembly of adhesive pili which is managed by PapD-like chaperones in Gram-negative bacteria, and (4) flagellar basal body-associated protein FliL: flagellum-specific transport system may be directly involved in the export of substrates into the central channel for flagellin transport. COTT also had Type II secretory pathway pseudopilin, PulG.

*6. Siderophore production for Fe uptake*

In response to extremely low concentrations of dissolved iron, some bacteria produce and express siderophores - small, high-affinity iron-chelating compounds which enable cells to acquire iron from the environment. COTT has one gene encoding 4'-phosphopantetheinyl transferase EntD, involved in siderophore biosynthesis, suggesting that siderophores are decisive for iron uptake under iron-limiting conditions of crude oil-contaminated soil. Additionally, siderophore production provides a competitive advantage to those bacteria capable of biosynthesis. Siderophores are shared secreted molecules that are energetically expensive to produce and as such, bacterial populations can opt to lose the ability to make these iron-binding products but still retain the ability for uptake. For the COTT strain, it was important that its genome be “in-charge” of its own siderophore production as several of its key redox reactions and regulatory proteins depended on iron [28].

*7. Nutrient rescue and transport*

Energy-coupling factor (ECF) transporters are members of the largest superfamily of membrane transport protein, the ATP-binding cassette (ABC) family of transporters, which are responsible for micronutrient uptake from the environment [29]. The ECF transporter can interact with different S components (or EcfS protein) and T/A/A′ components (or transmembrane domain, EcfT/A/A′ proteins; ECF module). The COTT genome alone had two ECFs, ECF transporter transmembrane protein EcfT (COG0619) and ECF transporter ATP-binding protein EcfA2 (COG1122).

*8. Salt stress*

During sudden increases in environmental salinity (salt shock) large amounts of inorganic ions enter the bacterial cell which interferes with intracellular ion homeostasis [30]. This disruption affects several metabolic processes, either directly or indirectly, as they are dependent on several inorganic ions e.g., potassium (K^+^), calcium (Ca^2+^), and magnesium (Mg^2+^). The magnesium transporter A (MgtA) (COG0474) is a P-type ATPase that is specialized to import Mg^2+^ into the cytoplasm. MgtA was only found in the COTT genome. Compatible solutes, or osmolytes, are low-molecular mass molecules that are readily soluble in water and can accumulate in high concentrations within the cell without affecting metabolism and other intra- cellular processes that serve to regulate the cell’s osmotic potential to maintain cell turgor and growth. [31].

The COTT genome contained the following genes that were present only in one other *C. oraminense* genome (either DSM 19055 or G3111 but not both): (1) ectoine hydroxylase-related dioxygenase/phytanoyl-CoA dioxygenase (PhyH) family, (2) ABC-type proline/glycine/betaine transport system, ATPase component, (3) ABC-type sugar transport system, permease component, (4) periplasmic glycine betaine/choline-binding (lipo)protein of an ABC-type transport system (an osmoprotectant binding protein), (5) osmotically-inducible protein OsmY, contains BON domain: osmY, was induced 8- to 10-fold by hyperosmotic stress [30], and (6) positive regulator of sigma E activity, rpoE (COG3086) which encodes a general stress response sigma transcription factor.

*9. Facultative respiration: aerobic/anaerobic generalized stress responses*

There are two main strategies employed by bacteria to manage oxidative stress [228-229]. The COTT genome has the following stress response-specific genes/enzymes: (1) the *rpoE* gene (COG3086), which encodes the general stress response sigma transcription factor, (2) ATP-dependent Clp protease adapter protein ClpS, ClpXP manages protein turn-over in response to heat shock and other stressors that damage proteins, (3) rubrerythrin, a non-heme metalloprotein that belongs to the ferritin-like superfamily that is involved in oxidative stress tolerance in anaerobic bacteria, (4) azurin which provides additional electron acceptors in redox reactions, (5) thioredoxin-related protein that may play a role in redox sensing and electron transfer, (6) ferredoxin (Fed), small proteins with highly negative redox potential that utilize their iron-sulfur cluster to serve as electron distributors in various metabolic pathways; Fed2-Fed9 are encoded by differently expressed genes and are low-abundant proteins that function in environmental stress tolerance; these proteins also participate in ferredoxin-glutaredoxin-thioredoxin crosstalk pathways that function to protect against oxidative and metal stresses, (7) bacterioferritin, Bfrs, whose function is to regulate iron homeostasis where it compartmentalizes iron when iron stores are very high and thus aid in enhancing resistance to oxidative stress and protect the reducing cell environment from unwanted Fe^3+^/Fe^2+^ redox cycling[32]; there is one bacterioferritin (cytochrome *b*1) gene that was only present in the COTT genome, (8) *cbb3*-type cytochrome oxidase and *cbb3*-type cytochrome oxidase subunit 3; *aa*_3_-type cytochrome *c* oxidase is commonly found in aerobes while the *cbb*_3_-type alternative cytochrome *c* oxidase is expressed mainly in strains that lack the *aa*_3_-type oxidase [33], (9) cytochrome *c5* peroxidase, (10) succinate dehydrogenase/fumarate reductase/fumarate hydratase, cytochrome *b* subunit: fumarate addition and hydroxylation are confirmed for anaerobic degradation of hydrocarbons [34], (11) carbon monoxide dehydrogenase (CODH) subunit G which is involved in CO oxidation and in aerobic and anaerobic pathways of carbon metabolism, (12) membrane-anchored ribosome-binding protein that inhibits bacterial growth in the stationary phase; ElaB/YqjD/DUF883 family: YqjD, is an inner membrane and ribosome binding protein expressed during the stationary growth phase; it is well-established cells that are oxygen-starved and/or found in the anaerobic stationary phase/non-growing state which renders these bacteria resistant to antimicrobials, (13) formyl-methanofuran dehydrogenase, subunit A; anaerobic organisms that convert methane to CO_2_ as a metabolic by-product in hypoxic conditions have this gene, (14) anaerobic selenocysteine-containing dehydrogenase that functions in anaerobic metabolism, and (15) hydration of NAD(P)H to NAD(P)HX, which constrains the function of several dehydrogenases, but this hydration is rectified by an ATP-dependent dehydratase and an epimerase; a deficiency in NADHX repair can result in mitochondrial dysfunction; COTT had one NAD(P)H-hydrate repair enzyme Nnr, NAD(P)H-hydrate epimerase domain. Such counteractive measures against nutrient stress, oxidative and nitrosative stress, cell envelope stress and other types of environmental stress serve to enhance bacterial survivability in growth-compromising conditions [35].

Castaño-Cerezo, et al. [36] reported that phosphotransacetylase (Pta) is abundant among fermentative anaerobes as it plays an integral role in acetate metabolism where it reversibly catalyzes the transfer of an acetyl group from acetyl phosphate to coenzyme A (CoA) to produce acetyl CoA and inorganic phosphate; Pta prevented accumulation of pyruvateacetyl-CoA, where pyruvate serves as an intermediate of central metabolism. There is one phosphotransacetylase (Pta)-encoding gene only found in the COTT genome and its encoded protein may serve as a pyruvate “gate-keeper”. This is important in COTT whose environmental carbon resource is high and therefore, carbon metabolism that ultimately leads to pyruvate synthesis must be regulated.

*10. DNA repair and fidelity of gene expression*

The COTT strain has a clear genomic emphasis on ensuring fidelity of gene expression and in maintaining the coding integrity of its genome because its environment is not forgiving to errors in the genome. In addition to tasking its genome with faithful replication and gene expression, COTT actively protected its genome stability from insertional mutagenesis by an invading phage or other transpositional and/or conjugative events by other bacteria. Several mechanisms were implied in COTT COG discovery and these are discussed below.

*10.1 DNA polymerase III*

DNA polymerase III is a replicative polymerase whose structure consists of a three-subunit core in addition to seven accessory subunits. Of these seven, *tau* and *gamma* are protein products of the *dna*X gene. Although the DNA polymerase III holoenzyme contains both, *tau* and *gamma*, only *tau* is essential which after proteolytic modification, can be substituted for the usual function(s) of *gamma* [37]. The COTT genome had two of these DNA polymerase III, *gamma*/*tau* subunit genes.

*10.2 Base excision repair*

DNA glycosylases function to protect genome integrity and provide base-specificity in the base excision repair pathway [38]. Bacterial AlkC and AlkD are DNA glycosylases whose substrates are cationic alkylated nucleobases. AlkD catalyzes excision of bulky lesions more commonly managed by nucleotide excision repair mechanism. In contrast, AlkC is specific for repairing small lesions e.g., N3-methyladenine (3mA) [39]. Only the COTT genome had AlkC. COTT and DSM 19055 both have a higher number of DNA repair genes than G311.

*10.3 Nucleotide excision repair*

ERCC excision repair 4 (excision repair cross-complementation group 4) forms the ERCC Endonuclease Catalytic Subunit. ERCC4 complexes with ERCC1 to direct 5' cleavage during nucleotide excision repair [40]. There was one ERCC4 and three endonuclease I encoding genes in the COTT genome.

*10.4 Double-strand break repair and mismatched repair*

The precise biological roles of exonucleases are difficult to delineate due to their functional redundancy [41]. RecBCD nucleases, originally termed exonuclease V or DNA exonucleases, hydrolyze the phosphodiester bonds in the DNA structure and as such, play important roles in DNA repair, including double-strand break (DSB) repair and mismatched repair (MMR) mechanisms [42], in addition to recombination and mutation prevention in all organisms. The COTT genome had one ATP-dependent exoDNAse (exonuclease V), alpha subunit, helicase superfamily I.

*10.5 Transcription and translation check points*

Transcription factors TFIIF and TFIIS are required for assembly of the transcription initiation complex and recruitment of RNA polymerase II [43]. The COTT genome had the TFIIS protein as well as the TFIIS with Zn-ribbon domain (DUF1610 family) which is essential for stimulation of RNA cleavage.

Nus, or N utilization substance proteins (NusA, NusB, NusE, and NusG), are a group of conserved bacterial transcription termination factors that function in transcription elongation, termination, anti-termination, and translation processes. The COTT genome had one transcription termination factor NusB. NusB deletion is conditionally lethal in *E. coli*, but the other Nus factors are essential. It is likely that the mechanisms of strict regulation of gene expression in COTT requires NusB termination factor.

Ribosomes must dissociate from stalled translation machinery. Removal of ribosomes facilitates decay of aberrant mRNA to ensure accurate protein synthesis [44]. The COTT genome had one stalled ribosome rescue protein Dom34, pelota family to improve the efficiency of translation and accuracy of protein synthesis.

Membrane-anchored ribosome-binding protein ElaB/YqjD/DUF883 family:YqjD, is an inner membrane- and ribosome-binding protein that functions to localize a part of the ribosome to the membrane during the stationary phase. Stress response sigma factor RpoS regulates this activity [45]. Only the COTT genome had one membrane-anchored ribosome-binding protein, of the ElaB/YqjD/DUF883 family which indicated a mechanism to stall translation to induce a state of temporary dormancy in response to otherwise lethal exposure to xenobiotics.

Bacterial initiation factor-2 (IF) 2 is a GTPase that stimulates binding of the initiator fMet-tRNAfMet to the 30S ribosomal subunit in assembly of the translation initiation complex. (IF) 2, therefore, contributes to maintaining the fidelity of translation initiation [46]. One copy of the translation initiation factor 2-encoded gene was present only in the COTT genome.

*10.6 DNA gyrase inhibitor*

Gyrases are topoisomerases that function to control the topological conformations of DNA. Multidrug resistance to gyrase inhibitors, e.g., fluoroquinolones, in pathogenic bacteria has arisen due to overuse of these drugs. The discovery and characterization of new antibacterial agents with novel mechanisms for DNA gyrase inhibition is important [47]. Only the COTT genome contained one gene encoding DNA gyrase inhibitor, GyrI (COG3449) and this should be a target for further exploration.

*10.7 Xenobiotic response element transcriptional regulator*

Xenobiotic-response element (XRE) proteins are involved in regulating genes for xenobiotic metabolism which is an adaptive function. It was also proposed that XREs comprise toxin–antitoxin (TA) type II systems. They appear to be one of the most important proteins involved in the control several diverse metabolic functions including gene regulation in response to DNA damage [48, 49]. The COTT genome had 11 copies of the gene encoding the transcriptional regulator containing the XRE-family HTH domain. COTT also had pgdA, peptidoglycan-N-acetylglucosamine deacetylase, which confers lysozyme resistance in the stationary phase of growth and has the potential to be developed as an antibiofilm therapeutic [50].

*10.8 Acetylation in gene activation*

There were 14 N-acetyltransferase, GNAT superfamily (includes histone acetyltransferase HPA2) genes in the COTT genome. It is purported that in the COTT genome multiple gene copies of GNAT, specifically HPA2, may be indicative of a high level of regulation of acetylation of lysine residues particularly in activation of gene expression. This is expected for organisms that can rapidly respond to changing environments. While GNATs are abundant and participate in several aspects of eukaryotic and prokaryotic physiology, the functions of many GNATs remain unknown [51].

*11. Stress-induced anti-social behavior of COTT*

The COTT genome contained several molecular signatures that appeared to enable it to outcompete and consume more nutritional resources according to the “resource ratio” model of competitive interactions [52].

Only the COTT genome harboured a gene encoding the Txe/YoeB family of bacterial toxins that forms part of the type II toxin-antitoxin system [53]. In the natural environment, these toxins may act in the selective killing of bacterial subpopulations during periods of nutritional or other stress. Type I and II systems aid in an apparent plasmid stabilization in bacterial populations [54]. Type II systems also afford bacteria the capacity to outcompete plasmids of the same incompatibility group [55]. Type II systems are maintained in the given bacterial population by horizontal gene transfer (HGT) [54] and are involved in the stabilization of large genomic fragments [56] and of integrative conjugative elements [54].

Restriction endonucleases (REs) serve as a primary defense mechanism against invading phages or other foreign DNA entering bacterial cells. Mrr (methylated adenine recognition and restriction) is a type IV restriction endonuclease that functions to recognize and cleave foreign methylated DNA. Mrr endonuclease activity results in double-strand breaks in the foreign DNA molecule [57]. There was one predicted gene encoding restriction endonuclease, Mrr-cat superfamily gene in the COTT genome.

Thiazolyl peptides (thiopeptides) belong to the group of macrocyclic peptide antibiotics which are highly modified heterocyclic peptides that are mainly active against Gram-positive bacteria [58]. COTT had two genes encoding thiopeptide-type bacteriocin biosynthesis protein (pfam14028 - Lant_dehydr_C) and demonstrated self-protection from its own thiopeptides through methylation of ribosome L11 via its ribosomal protein L11 methylase, PrmA.

*12. Arabinose efflux permease, MFS family – transmembrane protein*

The MFS superfamily includes uniporters, symporters or antiporters with diverse solute specificity for transporting small solutes in response to chemiosmotic ion gradients [59]. The COTT genome had 5 gene copies encoding MFS family permeases (COG0477) and 9 MFS transporters which reflected broad substrate specificity (simple sugars, oligosaccharides, inositols, drugs, amino acids, nucleosides, organophosphate esters and a large variety of organic and inorganic anions and cations) [59, 60].

L-arabinose is a source of carbon and energy. The L-arabinose operon (*ara* or *ara*BAD operon) is expressed for dedicated catabolism of arabinose to xylulose 5-phosphate (pentose phosphate pathway). The operon is regulated by positive and negative regulators. Four genes, *ara*A, *ara*B, *ara*C, and *ara*D, are associated with the uptake and conversion of L-arabinose inside the bacterial cell. *ara*C modulates the rate and amount of uptake of extracellular arabinose [61]. The *ara*C gene also interacts with histidine kinase sensors (perceives environmental stimuli) in signal transduction in TCS [62]. There were 63 copies in COTT of AraC-type DNA-binding domain and AraC-containing proteins – by far the highest of all the *C. oranimense* strains. COTT has the most *ara*C genes as an *ara* operon regulator (which indicates a high rate of arabinose catabolism) and the lowest number of arabinose efflux permease gene copies. Low efflux permease suggests arabinose cannot leave the cell easily.

Among 68 *Flavobacteria* species in OrthoDB, there were 92 genes with an IPR003313: AraC-type arabinose-binding/dimerization domain, as part of the structure of one of the transcription regulators of the *ara* operon; 23 species genomes contained a single copy of the gene and 19 species genomes contained multiple copies of the *araC* gene, all related through common ancestry. It was also estimated that the protein was not neutral and was under a moderate rate of evolutionary change (Fig. N 10).


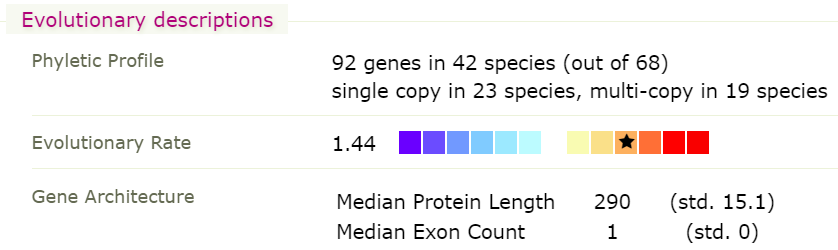


**Figure N10. Evolutionary analysis of AraC-type arabinose-binding/dimerization domain.** Relative rate is indicated by the position of the black star along the scale of slow (blue) to fast (red) rates.

Selective decomposition of plant polymers (e.g. lignin) results in pentose sugar accumulation in soil where arabinose and xylose are the dominant pentoses [63]. Apart from the major ligninolytic enzymes such as laccases and peroxidases, enzymes such as feruloyl esterase, quinone reductases, aryl-alcohol oxidase, lipases, xylanases and catechol 2, 3-dioxygenase facilitate degradation of lignocellulosic waste [64]. The latter three enzymes were found in higher abundance in the COTT genome compared to the other *C. oranimense*  genomes, some of which may show a higher affinity for arabinose as a natural substrate [65, 66]. Arabinose efflux effectors function to minimize accumulation of toxic sugar phosphates within the cell e.g., arabinose metabolic intermediate L-ribulose-5-phosphate, a build-up of which would become toxic to the cell [67]. It is possible that high levels of AraC may be important to relieving sugar-phosphate stress.

*13. Transformation and DNA uptake*

Transposases shift genes around in the genome, the result of which may be beneficial or detrimental to the bacterial cell. Transposition followed by integration into a genomic site with more relaxed sequence specificity can be a slow source of mutation generation, which may increase genetic diversity to drive adaptive evolution, and which may provide a genetic advantage to the COTT strain [68, 69].

The COTT genome had the transcriptional regulator of competence genes, TfoX/Sxy family. The tfoX (also called sxy) gene product functions to regulate DNA uptake in competent bacteria [70]. Environmental DNA accumulates after death and lysis of other cells. Competent bacteria utilize type IV pili to create transient pores in their membranes for DNA uptake into the cytoplasm [71]. Regulated DNA uptake mechanisms are widespread among bacteria and contributes to HGT.

Of the 9 genes that may have been acquired by COTT through HGT, only one gene encoding glycosyltransferase family 2 involved in cell wall biosynthesis was identified with high confidence (Pfam 97.23%) and whose donor was likely *Odoribacter splanchnicus* BIOML-A7 based on the available genomes in JGI. ABC-type lipoprotein export system ATPase subunit from *Chitinophaga ginsengisoli* DSM 18107 and ketosteroid isomerase-like protein from *Chitinophagaceae* sp. OAS944 were also putatively involved in HGT to COTT. All other proteins were hypothetical (Table N 11). The low number of genes potentially transferred to COTT genome through HGT is expected as a result of several control points to restrict foreign DNA uptake.

**Table N 11. Ten genes potentially acquired by COTT**

| **Class (Flavobacteria)** | **Gene Object Identifier** | **Product Name** | **From Gene** | **From Product** | **From Genome** | **pfam Hit** | **pfam description** | **pfam hit Percent Identity** |
| --- | --- | --- | --- | --- | --- | --- | --- | --- |
| **Bacteroidia** | 2945865941 | glycosyltransferase involved in cell wall biosynthesis | 2920487879 | glycosyltransferase involved in cell wall biosynthesis | *Odoribacter splanchnicus* BIOML-A7 | [pfam00535](http://pfam.xfam.org/family/PF00535) | Glycosyl transferase family 2 | 97.23 |
|  | 2945865943 | hypothetical protein | 647932335 | conserved hypothetical protein | *Prevotella* sp. F0039 | [pfam03414](http://pfam.xfam.org/family/PF03414) | Glycosyltransferase family 6 | 64.56 |
| Chitinophagia | 2945866898 | ABC-type lipoprotein export system ATPase subunit | 2730490008 | predicted ATPase | *Chitinophaga ginsengisoli* DSM 18107 | na | na | na |
|  | 2945866899 | hypothetical protein | 2694666065 | hypothetical protein | *Chitinophaga* sp. YR627 | na | na | na |
|  | 2945867432 | ketosteroid isomerase-like protein | 2931854865 | hypothetical protein | *Chitinophagaceae* sp. OAS944 | na | na | na |
| Cytophagia | 2945869357 | hypothetical protein | 2652754307 | hypothetical protein | *Sporocytophaga myxococcoides* PG-01 | [pfam19789](http://pfam.xfam.org/family/PF19789) | Family of unknown function (DUF6273) | 99.46 |
| Sphingobacteriia | 2945865942 | hypothetical protein | 2586209640 | EpsG family protein | *Pedobacter borealis* DSM 19626 | [pfam14897](http://pfam.xfam.org/family/PF14897) | EpsG family | 69.65 |
|  | 2945867223 | hypothetical protein | 2927659644 | hypothetical protein | *Sphingobacterium* sp. BIGb0165 | [pfam08928](http://pfam.xfam.org/family/PF08928) | Domain of unknown function (DUF1910) | 100 |
|  | 2945867394 | hypothetical protein | 2855561450 | hypothetical protein | *Pedobacter miscanthi* RS10 | na | na | na |
| **Phylum (Bacteroidetes)** |  |  |  |  |  |  |  |  |
| Apicomplexa | 2945869407 | hypothetical protein | 640363852 | hypothetical protein | *Plasmodium yoelii yoelii* 17XNL | na | na | na |

*14. Biofilm formation*

Only the COTT genome contained a gene-encoding GDP-D-mannose dehydratase which is the first step in 6-deoxyhexose biosynthesis in Gram-negative bacteria. 6-deoxysugars are critical subunits of cell surface glycans. Additionally, deoxysugars are also building blocks of lipopolysaccharides (LPSs), extracellular polysaccharides, glycoproteins, and some glycosylated secondary metabolites e.g., antibiotics [72]. The COTT genome encoded phosphomannomutase, an exopolysaccharide component of the *B. subtilis* biofilm [73], outer membrane murein-binding lipoprotein Lpp and outer membrane lipoprotein SlyB, a phosphopantetheinyl transferase where s*fp* encodes a phosphopantetheinyl transferase that activates the first three subunits (SrfABC) of surfactin synthetase [74]. Surfactin and a peptide signal, ComX, both contribute to biofilm formation in *Bacillus* [75-77]. Table N12 summarizes the genes and their encoded proteins predicted to have an adaptive function in the survival of COTT in soil chronically polluted with crude oil; these genes were only found in the COTT genome.

**Table N12. Summary of genes and their encoded proteins predicted to have an adaptive function in the survival of COTT in soil chronically polluted with crude oil**

| **System** | **COG/KO ID** | **COG/KO Name** | **Gene count** |
| --- | --- | --- | --- |
| **Motif repeats** | COG0457 | Tetratricopeptide (TPR) repeat | 16 |
|  | COG4886 | Leucine-rich repeat (LRR) protein | 3 |
| **Nutrient transport** | COG1226 | Voltage-gated potassium channel Kch | 2 |
|  | COG1178 | ABC-type Fe3+ transport system, permease component | 1 |
|  | COG2215 | ABC-type nickel/cobalt efflux system, permease component RcnA | 1 |
|  | COG1175 | ABC-type sugar transport system, permease component | 1 |
|  | COG1118 | ABC-type sulfate/molybdate transport systems, ATPase component | 1 |
|  | COG1277 | ABC-type transport system involved in multi-copper enzyme maturation, permease component | 3 |
|  | KO:K16267 | zipB; zinc and cadmium transporter | 2 |
|  | COG1122 | Energy-coupling factor transporter ATP-binding protein EcfA2 | 1 |
|  | COG0619 | Energy-coupling factor transporter transmembrane protein EcfT | 1 |
| **Salt tolerance** | COG5285 | Ectoine hydroxylase-related dioxygenase, phytanoyl-CoA dioxygenase (PhyH) family | 2 |
|  | COG2823 | Osmotically-inducible protein OsmY, contains BON domain | 1 |
|  | COG1732 | Periplasmic glycine betaine/choline-binding (lipo) osmoprotectant binding protein | 1 |
|  | KO:K03802 | cphA; osmoprotectant transport system ATP-binding protein | 1 |
|  | KO:K03809 | wrbA; osmoprotectant transport system permease protein | 1 |
| **Lipid bilayer** | KO:K01256 | pepN; cardiolipin synthase A/B | 1 |
|  | KO:K04773 | sppA; phosphatidylglycerol---prolipoprotein diacylglyceryl transferase | 2 |
|  | COG1376 | Lipoprotein-anchoring transpeptidase ErfK/SrfK | 1 |
| **Genome repair, expression and maintenance of integrity** | COG2812 | DNA polymerase III, gamma/tau subunits | 2 |
|  | COG3391 | DNA-binding beta-propeller fold protein YncE | 2 |
|  | COG3086 | Positive regulator of sigma E activity | 2 |
|  | COG0507 | ATP-dependent exoDNAse (exonuclease V), alpha subunit, helicase superfamily I | 1 |
|  | COG1758 | DNA-directed RNA polymerase, subunit K/omega | 1 |
|  | COG1594 | DNA-directed RNA polymerase, subunit M/Transcription elongation factor TFIIS | 1 |
|  | COG1996 | DNA-directed RNA polymerase, subunit RPC12/RpoP, contains C4-type Zn-finger | 1 |
|  | COG1537 | Stalled ribosome rescue protein Dom34, pelota family | 1 |
|  | COG4290 | Guanyl-specific ribonuclease Sa | 1 |
|  | COG3449 | DNA gyrase inhibitor GyrI | 1 |
|  | COG4227 | Antirestriction protein ArdC | 1 |
|  | COG0258 | 5'-3' exonuclease | 1 |
|  | COG0724 | RNA recognition motif (RRM) domain | 1 |
|  | COG4335 | 3-methyladenine DNA glycosylase AlkC | 1 |
|  | COG0582 | Integrase | 2 |
|  | COG2356 | Endonuclease I | 3 |
|  | COG4290 | Guanyl-specific ribonuclease Sa | 1 |
|  | COG0130 | tRNA U55 pseudouridine synthase TruB, may also work on U342 of tmRNA | 1 |
|  | COG1948 | ERCC4-type nuclease | 1 |
|  | KO:K19048 | symE; toxic protein SymE | 1 |
|  | COG2816 | NADH pyrophosphatase NudC, Nudix superfamily | 1 |
| **Anaerobic lifestyle** | COG4314 | Nitrous oxide reductase accessory protein NosL | 2 |
|  | COG0243 | Anaerobic selenocysteine-containing dehydrogenase | 1 |
|  | COG3427 | Carbon monoxide dehydrogenase subunit G | 1 |
|  | KO:K21562 | flp; CRP/FNR family transcriptional regulator, anaerobic regulatory protein | 1 |
|  | COG1633 | Rubrerythrin | 1 |
|  | COG2009 | Succinate dehydrogenase/fumarate reductase, cytochrome b subunit | 2 |
|  | COG1229 | Formylmethanofuran dehydrogenase subunit A | 1 |
| **Multiple cellular processes** | COG0454 | N-acetyltransferase, GNAT superfamily (includes histone acetyltransferase HPA2) | 14 |
|  |  |  |  |
| **Signalling** | COG3920 | Two-component sensor histidine kinase, HisKA and HATPase domains | 1 |
|  | COG3706 | Two-component response regulator, PleD family, consists of two REC domains and a diguanylate cyclase (GGDEF) domain | 2 |
|  | COG4753 | Two-component response regulator, YesN/AraC family, consists of REC and AraC-type DNA-binding domains | 2 |
|  | KO:K19611 | fepA, pfeA, iroN, pirA; ferric enterobactin receptor- iron acquisition | 1 |
|  | KO:K18940 | arlS; two-component system, OmpR family, sensor histidine kinase ArlS | 1 |
|  | KO:K18941 | arlR; two-component system, OmpR family, sensor histidine kinase ArlR | 1 |
|  | KO:K16692 | etk-wzc; tyrosine-protein kinase Etk/Wzc | 1 |
|  | COG2201 | Chemotaxis response regulator CheB, contains REC and protein-glutamate methylesterase domains | 1 |
|  | KO:K13566 | NIT2, yafV; two-component system, LytTR family, response regulator | 2 |
|  | KO:K13572 | pafB; two-component system, LytTR family, response regulator LytT | 4 |
|  | KO:K13598 | ntrY' two-component system, LytTR family, sensor kinase | 6 |
|  | COG4537 | Competence protein ComGC | 1 |
|  | COG1555 | DNA uptake protein ComE and related DNA-binding proteins | 1 |
|  | KO:K13566 | NIT2, yafV; two-component system, LytTR family, response regulator | 2 |
|  | KO:K13572 | pafB; two-component system, LytTR family, response regulator LytT | 4 |
|  | KO:K13598 | ntrY; two-component system, LytTR family, sensor kinase | 6 |
| **Siderophore biosynthesis** | COG2977 | 4'-phosphopantetheinyl transferase EntD (siderophore biosynthesis) | 1 |
|  | KO:K00384 | trx, TRR; 4'-phosphopantetheinyl transferase | 1 |
|  | KO:K01933 | furM; ferric enterobactin receptor | 1 |
| **Exopolysaccharide/ Biofilm formation** | COG5309 | Exo-beta-1,3-glucanase, GH17 family | 1 |
|  | COG0248 | Exopolyphosphatase/pppGpp-phosphohydrolase | 1 |
|  | COG4632 | Exopolysaccharide biosynthesis protein related to N-acetylglucosamine-1-phosphodiester alpha-N-acety... | 1 |
|  | COG3979 | Chitodextrinase | 1 |
|  | KO:K20968 | exsA; exoenzyme S synthesis regulatory protein | 1 |
|  | KO:K21449 | ata, sadA, emaA; trimeric autotransporter adhesin | 1 |
|  | KO:K19294 | algI; alginate O-acetyltransferase complex protein | 1 |
|  | KO:K16710 | wcaK, amsJ; colanic acid/amylovoran biosynthesis protein WcaK/AmsJ | 2 |
|  | COG3206 | Uncharacterized protein involved in exopolysaccharide biosynthesis | 3 |
| **Flagellar elements/quorum sensing** | COG1580 | Flagellar basal body-associated protein FliL | 1 |
|  | COG1360 | Flagellar motor protein MotB | 1 |
|  | COG4965 | Flp pilus assembly protein TadB | 1 |
|  | COG0455 | MinD-like ATPase involved in chromosome partitioning or flagellar assembly | 1 |
|  | COG3121 | P pilus assembly protein, chaperone PapD | 1 |
|  | COG2165 | Type II secretory pathway, pseudopilin PulG | 1 |

**References**

1. Baumgartner JT, Habeeb Mohammad TS, Czub MP, Majorek KA, Arolli X, Variot C, et al. Gcn5-related N-acetyltransferases (GNATs) with a catalytic serine residue can play ping-pong too. Front Mol Biosci. 2021;8; doi: 10.3389/fmolb.2021.646046.

2. Hentchel Kristy L, Escalante-Semerena Jorge C. Acylation of biomolecules in prokaryotes: a widespread strategy for the control of biological function and metabolic stress. Microbiol Mol Biol Rev. 2015;79(3):321-46; doi: 10.1128/MMBR.00020-15.

3. Mitchell AM, Silhavy TJ. Envelope stress responses: balancing damage repair and toxicity. Nat Rev Microbiol. 2019;17(7):417-28; doi: 10.1038/s41579-019-0199-0.

4. Hews CL, Cho T, Rowley G, Raivio TL. Maintaining integrity under stress: envelope stress response regulation of pathogenesis in Gram-negative bacteria. Front Cell Infect Microbiol. 2019;9; doi: 10.3389/fcimb.2019.00313.

5. Chautrand T, Souak D, Chevalier S, Duclairoir-Poc C. Gram-negative bacterial envelope homeostasis under oxidative and nitrosative stress. Microorganisms. 2022;10(5):924.

6. Rowley G, Spector M, Kormanec J, Roberts M. Pushing the envelope: extracytoplasmic stress responses in bacterial pathogens. Nat Rev Microbiol. 2006;4(5):383-94; doi: 10.1038/nrmicro1394.

7. Kwon E, Kim DY, Gross CA, Gross JD, Kim KK. The crystal structure *Escherichia coli* Spy. Protein Sci. 2010;19(11):2252-9; doi: https://doi.org/10.1002/pro.489.

8. Kim H, Wu K, Lee C. Stress-responsive periplasmic chaperones in bacteria. Front Mol Biosci. 2021;8; doi: 10.3389/fmolb.2021.678697.

9. Kamennaya NA, Geraki K, Scanlan DJ, Zubkov MV. Accumulation of ambient phosphate into the periplasm of marine bacteria is proton motive force dependent. Nat Commun. 2020;11(1):2642; doi: 10.1038/s41467-020-16428-w.

10. Vergalli J, Bodrenko IV, Masi M, Moynié L, Acosta-Gutiérrez S, Naismith JH, et al. Porins and small-molecule translocation across the outer membrane of Gram-negative bacteria. Nat Rev Microbiol. 2020;18(3):164-76; doi: 10.1038/s41579-019-0294-2.

11. Chhabra S, Brazil D, Morrissey J, Burke JI, O'Gara F, N. Dowling D. Characterization of mineral phosphate solubilization traits from a barley rhizosphere soil functional metagenome. MicrobiologyOpen. 2013;2(5):717-24; doi: https://doi.org/10.1002/mbo3.110.

12. Ponsin V, Mouloubou OR, Prudent P, Höhener P. Does phosphate enhance the natural attenuation of crude oil in groundwater under defined redox conditions? J Contam Hydrol. 2014;169:4-18; doi: https://doi.org/10.1016/j.jconhyd.2014.04.003.

13. Yoshida M, Ishihama A, Yamamoto K. Cross talk in promoter recognition between six NarL-family response regulators of *Escherichia coli* two-component system. Genes Cells. 2015;20(7):601-12; doi: https://doi.org/10.1111/gtc.12251.

14. Galperin MY. Bacterial signal transduction network in a genomic perspective. Environ Microbiol. 2004;6(6):552-67; doi: https://doi.org/10.1111/j.1462-2920.2004.00633.x.

15. Baykov AA, Tuominen HK, Lahti R. The CBS domain: a protein module with an emerging prominent role in regulation. ACS Chem Biol. 2011;6(11):1156-63; doi: 10.1021/cb200231c.

16. Galperin MY. Structural classification of bacterial response regulators: diversity of output domains and domain combinations. J Bacteriol. 2006;188(12):4169-82; doi: doi:10.1128/JB.01887-05.

17. Shiomi D, Zhulin IB, Homma M, Kawagishi I. Dual recognition of the bacterial chemoreceptor by chemotaxis-specific domains of the CheR methyltransferase J Biol Chem. 2002;277(44):42325-33; doi: 10.1074/jbc.M202001200.

18. Wunsch P, Herb M, Wieland H, Schiek Ulrike M, Zumft Walter G. Requirements for CuA and Cu-S center assembly of nitrous oxide reductase deduced from complete periplasmic enzyme maturation in the nondenitrifier *Pseudomonas putida*. J Bacteriol. 2003;185(3):887-96; doi: 10.1128/JB.185.3.887-896.2003.

19. Prasser B, Schöner L, Zhang L, Einsle O. The copper chaperone nosl forms a heterometal site for cu delivery to nitrous oxide reductase. Angew Chem Int Ed. 2021;60(34):18810-4; doi: https://doi.org/10.1002/anie.202106348.

20. Stauffert M, Cravo-Laureau C, Duran R. Structure of hydrocarbonoclastic nitrate-reducing bacterial communities in bioturbated coastal marine sediments. FEMS Microbiol Ecol. 2014;89(3):580-93; doi: 10.1111/1574-6941.12359.

21. Zhao Y, Chen W, Wen D. The effects of crude oil on microbial nitrogen cycling in coastal sediments. Environ Int. 2020;139:105724; doi: https://doi.org/10.1016/j.envint.2020.105724.

22. Muramoto K, Macnab RM. Deletion analysis of MotA and MotB, components of the force-generating unit in the flagellar motor of *Salmonella*. Mol Microbiol. 1998;29(5):1191-202; doi: https://doi.org/10.1046/j.1365-2958.1998.00998.x.

23. Nguyen CC, Saier MH. Structural and phylogenetic analysis of the MotA and MotB families of bacterial flagellar motor proteins. Res Microbiol. 1996;147(5):317-32; doi: https://doi.org/10.1016/0923-2508(96)84707-3.

24. Sharma P, Gupta SK, Diene SM, Rolain J-M. Whole-genome sequence of *Chryseobacterium oranimense*, a colistin-resistant bacterium isolated from a cystic fibrosis patient in france. Antimicrob Agents Chemother. 2015;59(3):1696-706; doi: doi:10.1128/AAC.02417-14.

25. Hantsis-Zacharov E, Shakéd T, Senderovich Y, Halpern M. *Chryseobacterium oranimense* sp. nov., a psychrotolerant, proteolytic and lipolytic bacterium isolated from raw cow’s milk. Int J Syst Evol Microbiol. 2008;58(11):2635-9; doi: https://doi.org/10.1099/ijs.0.65819-0.

26. Dahal RH, Chaudhary DK, Kim D-U, Pandey RP, Kim J. *Chryseobacterium antibioticum* sp. nov. with antimicrobial activity against Gram-negative bacteria, isolated from Arctic soil. J Antibiot Res. 2021;74(2):115-23; doi: 10.1038/s41429-020-00367-1.

27. Meng D, Liu Y-L, Li R-R, Gu P-F, Fan X-Y, Huang Z-S, et al. *Chryseobacterium binzhouense* sp. nov., isolated from activated sludge. Int J Syst Evol Microbiol. 2020;70(1):618-23; doi: https://doi.org/10.1099/ijsem.0.003800.

28. Frawley ER, Fang FC. The ins and outs of bacterial iron metabolism. Mol Microbiol. 2014;93(4):609-16; doi: https://doi.org/10.1111/mmi.12709.

29. Erkens GB, Majsnerowska M, ter Beek J, Slotboom DJ. Energy coupling factor-type ABC transporters for vitamin uptake in prokaryotes. Biochem. 2012;51(22):4390-6; doi: 10.1021/bi300504v.

30. Yim HH, Villarejo M. osmY, a new hyperosmotically inducible gene, encodes a periplasmic protein in *Escherichia coli*. J Bacteriol. 1992;174(11):3637-44; doi: doi:10.1128/jb.174.11.3637-3644.1992.

31. Kirsch F, Klähn S, Hagemann M. Salt-regulated accumulation of the compatible solutes sucrose and glucosylglycerol in *Cyanobacteria* and its biotechnological potential. Front Microbiol. 2019;10; doi: 10.3389/fmicb.2019.02139.

32. Rivera M. Bacterioferritin: structure, dynamics, and protein–protein interactions at play in iron storage and mobilization. Acc Chem Res. 2017;50(2):331-40; doi: 10.1021/acs.accounts.6b00514.

33. Toledo-Cuevas M, Barquera B, Gennis RB, Wikström M, Garcı́a-Horsman JA. The cbb3-type cytochrome c oxidase from *Rhodobacter sphaeroides*, a proton-pumping heme-copper oxidase. Biochim Biophys Acta. 1998;1365(3):421-34; doi: https://doi.org/10.1016/S0005-2728(98)00095-4.

34. Wartell B, Boufadel M, Rodriguez-Freire L. An effort to understand and improve the anaerobic biodegradation of petroleum hydrocarbons: a literature review. Int Biodeterior Biodegrad. 2021;157:105156; doi: https://doi.org/10.1016/j.ibiod.2020.105156.

35. Poole K. Stress responses as determinants of antimicrobial resistance in Gram-negative bacteria. Trends Microbiol. 2012;20(5):227-34; doi: https://doi.org/10.1016/j.tim.2012.02.004.

36. Castaño-Cerezo S, Pastor JM, Renilla S, Bernal V, Iborra JL, Cánovas M. An insight into the role of phosphotransacetylase (pta) and the acetate/acetyl-CoA node in *Escherichia coli*. Microbial Cell Factories. 2009;8(1):54; doi: 10.1186/1475-2859-8-54.

37. Blinkova A, Hervas C, Stukenberg PT, Onrust R, O'Donnell ME, Walker JR. The *Escherichia coli* DNA polymerase III holoenzyme contains both products of the dnaX gene, tau and gamma, but only tau is essential. J Bacteriol. 1993;175(18):6018-27; doi: 10.1128/jb.175.18.6018-6027.1993.

38. Mullins EA, Rodriguez AA, Bradley NP, Eichman BF. Emerging roles of DNA glycosylases and the base excision repair pathway. Trends Biochem Sci. 2019;44(9):765-81; doi: https://doi.org/10.1016/j.tibs.2019.04.006.

39. Shi R, Mullins EA, Shen X-X, Lay KT, Yuen PK, David SS, et al. Selective base excision repair of DNA damage by the non-base-flipping DNA glycosylase AlkC. EMBO J. 2018;37(1):63-74; doi: https://doi.org/10.15252/embj.201797833.

40. Manandhar M, Boulware KS, Wood RD. The ERCC1 and ERCC4 (XPF) genes and gene products. Gene. 2015;569(2):153-61; doi: https://doi.org/10.1016/j.gene.2015.06.026.

41. Mason PA, Cox LS. The role of DNA exonucleases in protecting genome stability and their impact on ageing. AGE. 2012;34(6):1317-40; doi: 10.1007/s11357-011-9306-5.

42. Lovett Susan T. The DNA exonucleases of *Escherichia coli*. EcoSal Plus. 2011;4(2); doi: 10.1128/ecosalplus.4.4.7.

43. Kim B, Nesvizhskii AI, Rani PG, Hahn S, Aebersold R, Ranish JA. The transcription elongation factor TFIIS is a component of RNA polymerase II preinitiation complexes. Proceedings of the National Academy of Sciences. 2007;104(41):16068-73; doi: 10.1073/pnas.0704573104.

44. Buskirk AR, Green R. Ribosome pausing, arrest and rescue in bacteria and eukaryotes. Philos Trans Royal Soc B: Biol Sci. 2017;372(1716):20160183; doi: 10.1098/rstb.2016.0183.

45. Yoshida H, Maki Y, Furuike S, Sakai A, Ueta M, Wada A. YqjD is an inner membrane protein associated with stationary-phase ribosomes in *Escherichia coli*. J Bacteriol. 2012;194(16):4178-83; doi: 10.1128/JB.00396-12.

46. Caban K, Pavlov M, Ehrenberg M, Gonzalez RL. A conformational switch in initiation factor 2 controls the fidelity of translation initiation in bacteria. Nat Commun. 2017;8(1):1475; doi: 10.1038/s41467-017-01492-6.

47. Collin F, Karkare S, Maxwell A. Exploiting bacterial DNA gyrase as a drug target: current state and perspectives. Appl Microbiol Biotechnol. 2011;92(3):479-97; doi: 10.1007/s00253-011-3557-z.

48. Liu Y, Zhou J, Omelchenko MV, Beliaev AS, Venkateswaran A, Stair J, et al. Transcriptome dynamics of *Deinococcus radiodurans* recovering from ionizing radiation. Proceedings of the National Academy of Sciences. 2003;100(7):4191-6; doi: 10.1073/pnas.0630387100.

49. Novichkov PS, Kazakov AE, Ravcheev DA, Leyn SA, Kovaleva GY, Sutormin RA, et al. RegPrecise 3.0 - A resource for genome-scale exploration of transcriptional regulation in bacteria. BMC Genom. 2013;14(1):745; doi: 10.1186/1471-2164-14-745.

50. Rouchon Candace N, Harris J, Zubair-Nizami Z, Weinstein Arielle J, Roky M, Frank Kristi L. The cationic antimicrobial peptide activity of lysozyme reduces viable *Enterococcus faecalis* cells in biofilms. Antimicrob Agents Chemother. 2022;66(5):e02339-21; doi: 10.1128/aac.02339-21.

51. Hu LI, Lima BP, Wolfe AJ. Bacterial protein acetylation: the dawning of a new age. Mol Microbiol. 2010;77(1):15-21; doi: https://doi.org/10.1111/j.1365-2958.2010.07204.x.

52. Hibbing ME, Fuqua C, Parsek MR, Peterson SB. Bacterial competition: surviving and thriving in the microbial jungle. Nat Rev Microbiol. 2010;8(1):15-25; doi: 10.1038/nrmicro2259.

53. Leplae R, Geeraerts D, Hallez R, Guglielmini J, Drèze P, Van Melderen L. Diversity of bacterial type II toxin–antitoxin systems: a comprehensive search and functional analysis of novel families. Nucleic Acids Res. 2011;39(13):5513-25; doi: 10.1093/nar/gkr131.

54. Wozniak RAF, Waldor MK. A toxin–antitoxin system promotes the maintenance of an integrative conjugative element. PLoS Genet. 2009;5(3):e1000439; doi: 10.1371/journal.pgen.1000439.

55. Cooper TF, Heinemann JA. Postsegregational killing does not increase plasmid stability but acts to mediate the exclusion of competing plasmids. Proceedings of the National Academy of Sciences. 2000;97(23):12643-8; doi: 10.1073/pnas.220077897.

56. Szekeres S, Dauti M, Wilde C, Mazel D, Rowe-Magnus DA. Chromosomal toxin–antitoxin loci can diminish large-scale genome reductions in the absence of selection. Mol Microbiol. 2007;63(6):1588-605; doi: https://doi.org/10.1111/j.1365-2958.2007.05613.x.

57. Bourges AC, Torres Montaguth OE, Tadesse W, Labesse G, Aertsen A, Royer CA, et al. An oligomeric switch controls the Mrr-induced SOS response in *E. coli*. DNA Repair. 2021;97:103009; doi: https://doi.org/10.1016/j.dnarep.2020.103009.

58. Engelhardt K, Degnes KF, Zotchev SB. Isolation and characterization of the gene cluster for biosynthesis of the thiopeptide antibiotic TP-1161. Appl Environ Microbiol. 2010;76(21):7093-101; doi: doi:10.1128/AEM.01442-10.

59. Pao Stephanie S, Paulsen Ian T, Saier Milton H. Major facilitator superfamily. Microbiol Mol Biol Rev. 1998;62(1):1-34; doi: 10.1128/MMBR.62.1.1-34.1998.

60. Reddy VS, Shlykov MA, Castillo R, Sun EI, Saier Jr MH. The major facilitator superfamily (MFS) revisited. FEBS J. 2012;279(11):2022-35; doi: https://doi.org/10.1111/j.1742-4658.2012.08588.x.

61. Schleif R. AraC protein, regulation of the l-arabinose operon in *Escherichia coli*, and the light switch mechanism of AraC action. FEMS Microbiol Rev. 2010;34(5):779-96; doi: 10.1111/j.1574-6976.2010.00226.x.

62. Galperin MY. A census of membrane-bound and intracellular signal transduction proteins in bacteria: Bacterial IQ, extroverts and introverts. BMC Microbiol. 2005;5(1):35; doi: 10.1186/1471-2180-5-35.

63. Gunina A, Kuzyakov Y. Sugars in soil and sweets for microorganisms: review of origin, content, composition and fate. Soil Biol Biochem. 2015;90:87-100; doi: https://doi.org/10.1016/j.soilbio.2015.07.021.

64. Kumar A, Chandra R. Ligninolytic enzymes and its mechanisms for degradation of lignocellulosic waste in environment. Heliyon. 2020;6(2); doi: 10.1016/j.heliyon.2020.e03170.

65. Hunt CJ, Antonopoulou I, Tanksale A, Rova U, Christakopoulos P, Haritos VS. Insights into substrate binding of ferulic acid esterases by arabinose and methyl hydroxycinnamate esters and molecular docking. Sci Rep. 2017;7(1):17315; doi: 10.1038/s41598-017-17260-x.

66. Xu Z, He H, Zhang S, Guo T, Kong J. Characterization of feruloyl esterases produced by the four *Lactobacillus* species: *L. amylovorus*, *L. acidophilus*, *L. farciminis* and *L. fermentum*, Isolated from Ensiled Corn Stover. Front Microbiol. 2017;8; doi: 10.3389/fmicb.2017.00941.

67. Koita K, Rao CV. Identification and analysis of the putative pentose sugar efflux transporters in *Escherichia coli*. PLOS ONE. 2012;7(8):e43700; doi: 10.1371/journal.pone.0043700.

68. Hickman Alison B, Dyda F. Mechanisms of DNA transposition. Microbiol Spectr. 2015;3(2):3.2.12; doi: 10.1128/microbiolspec.MDNA3-0034-2014.

69. Curcio MJ, Derbyshire KM. The outs and ins of transposition: from Mu to Kangaroo. Nat Rev Mol Cell Biol. 2003;4(11):865-77; doi: 10.1038/nrm1241.

70. Yamamoto S, Izumiya H, Mitobe J, Morita M, Arakawa E, Ohnishi M, et al. Identification of a chitin-induced small RNA That regulates translation of the tfoX gene, encoding a positive regulator of natural competence in *Vibrio cholerae*. J Bacteriol. 2011;193(8):1953-65; doi: 10.1128/JB.01340-10.

71. Solomon JM, Grossman AD. Who's competent and when: regulation of natural genetic competence in bacteria. Trends Genet. 1996;12(4):150-5; doi: https://doi.org/10.1016/0168-9525(96)10014-7.

72. Mäki M, Renkonen R. Biosynthesis of 6-deoxyhexose glycans in bacteria. Glycobiology. 2004;14(3):1R-15R; doi: 10.1093/glycob/cwh040.

73. Marvasi M, Visscher PT, Casillas Martinez L. Exopolymeric substances (EPS) from *Bacillus subtilis*: polymers and genes encoding their synthesis. FEMS Microbiol Lett. 2010;313(1):1-9; doi: 10.1111/j.1574-6968.2010.02085.x.

74. Quadri LEN, Weinreb PH, Lei M, Nakano MM, Zuber P, Walsh CT. Characterization of Sfp, a *Bacillus subtilis* phosphopantetheinyl transferase for peptidyl carrier protein domains in peptide synthetases. Biochem. 1998;37(6):1585-95; doi: 10.1021/bi9719861.

75. Bindel Connelly M, Young Glenn M, Sloma A. Extracellular proteolytic activity plays a central role in swarming motility in *Bacillus subtilis*. J Bacteriol. 2004;186(13):4159-67; doi: 10.1128/JB.186.13.4159-4167.2004.

76. Hofemeister J, Conrad B, Adler B, Hofemeister B, Feesche J, Kucheryava N, et al. Genetic analysis of the biosynthesis of non-ribosomal peptide- and polyketide-like antibiotics, iron uptake and biofilm formation by *Bacillus subtilis* A1/3. Mol Genet Genomics. 2004;272(4):363-78; doi: 10.1007/s00438-004-1056-y.

77. López D, Vlamakis H, Losick R, Kolter R. Paracrine signaling in a bacterium. Genes Dev. 2009;23(14):1631-8; doi: 10.1101/gad.1813709.
