# Supplementary material for: Genome features of a novel hydrocarbonoclastic Chryseobacterium oranimense strain and its comparison to bacterial oil-degraders and to other C. oranimense strains
Source: DNA Res. 2023 Nov 11;30(6):dsad025. doi: 10.1093/dnares/dsad025 (PMC10710014; doi:10.1093/dnares/dsad025)
Supplement: dsad025_suppl_Supplementary_File_S4 [file dsad025_suppl_supplementary_file_s4.docx]

**Additional File 4: Supplementary Method**

**DNA qualification**

Novogene utilizes two major QC methods for DNA sample qualification:

(1) Agarose gel electrophoresis analysis for DNA purity and integrity;

(2) Qubit® 3.0 flurometer quantitation for accurate measurement of DNA

concentration.

Sample DNA with total amount of more than 500 ng was qualified for library construction.

**Library construction**

The genomic DNA of each sample was randomly sheared into short fragments of about 350 bp respectively. The obtained fragments were subjected to library construction using the NEBNext® DNA Library Prep Kit, following the instructions strictly. Briefly, as followed by end repairing, dA-tailing, and further ligation with NEBNext adapter, the required fragments (in 300-500 bp size) were PCR enriched by P5 and indexed P7 oligos. After purification and subsequent quality check, the library is ready for sequencing. The experimental procedures of DNA library preparation are shown in Figure S1.


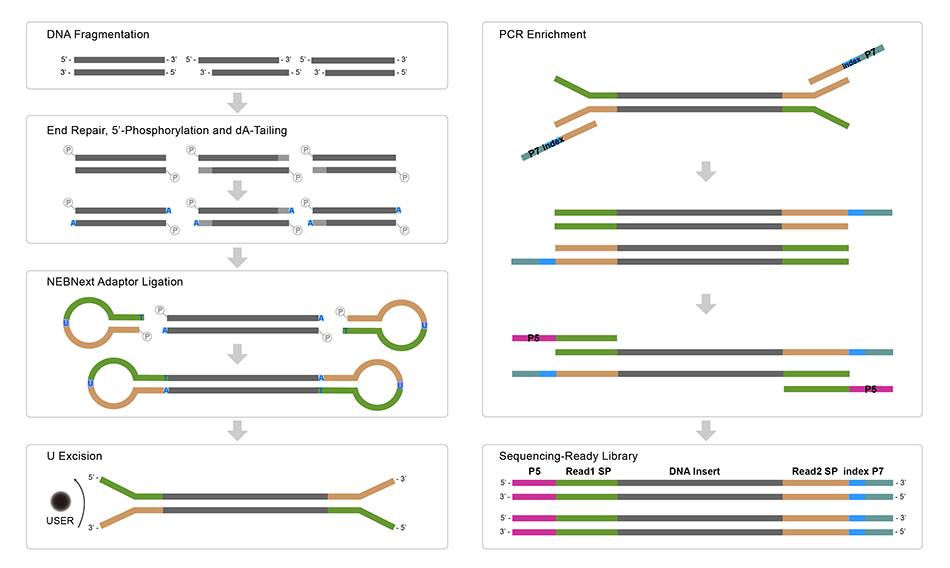
**Figure S1. Experimental procedures of library preparation.**

**Library quality control**

To check the prepared DNA libraries, Qubit 2.0 fluorometer was firstly used to determine the concentration of the library. After dilution to 1 ng/ul, the Agilent 2100 bioanalyzer was used to assess the insert size. Finally the quantitative real-time PCR (qPCR) was performed to detect the effective concentration of each library. If the library with appropriate insert size has an effective concentration (more than 2 nM), the constructed libraries are qualified and ready for Illumina high-throughput sequencing.

**High-throughput DNA sequencing**

The qualified DNA libraries were pooled according to their effective concentration as well as the expected data production. Pair-end sequencing were performed on a high-throughput Illumina sequencing platform (e.g. Illumina HiSeq/NovaSeq), with the read length of PE150 bp at each end. High-quality reads were obtained after quality control was performed in-house by Novogene.

**Bioinformatics analysis procedures**

The bioinformatic analysis procedures were as follows:

    (1) Quality control of raw sequencing data for clean data filtration;

    (2) Mapping clean reads to reference genome;

    (3) SNP, InDel, SV and CNV detection and annotation according to the mapping

results.

**Quality control**

Raw data

The original sequencing data acquired by high-throughput sequencing platforms recorded in image files are firstly transformed to sequence reads by base calling with the CASAVA software.

Sequencing data quality control

If the sequencing error rate is represented by e and Illumina sequencing quality by Q_phred_, the quality score of a base (Phred score) is calculated by the following equation: Q_phred_=-10log_10_(e). The correspondence relationship between Illunima sequencing quality and Phred score in base calling by Casava version 1.8 is listed as follows:

**Table S1. Sequencing error rate and corresponding base quality value.**

| **Phred score** | **Error Rate** | **Correct Rate** | **Q-score** |
| --- | --- | --- | --- |
| 10 | 1/10 | 90% | Q10 |
| 20 | 1/100 | 99% | Q20 |
| 30 | 1/1000 | 99.9% | Q30 |
| 40 | 1/10000 | 99.99% | Q40 |

Sequencing quality distribution was examined over the full length of all sequences, to detect any sites (base positions) with an unusually low sequencing quality, where incorrect bases may be incorporated at abnormally high levels.

Distribution of sequencing errors

Sequencing error rate distribution was examined over the full length of all sequences, to detect any sites (base positions) with an unusually high error rate, where incorrect bases may be incorporated at abnormally high levels.

Sequencing data filtration

Raw data obtained from sequencing contains adapter contamination and low-quality reads. These sequencing artifacts may increase the complexity of further analyses, so quality control steps were utilized to remove them. Consequently, all the further analyses were based on the clean reads. The quality control steps were as follows:

      (1) Discard the paired reads when either read contains adapter contamination;
      (2) Discard the paired reads when uncertain nucleotides (N) constitute more than

10 percent of either read;

(3) Discard the paired reads when low quality nucleotides (base quality less than 5,

Q ≤ 5) constitute more than 50 percent of either read.

**Mapping statistics**

The effective sequencing data were aligned with the reference sequences through BWA [1] software (parameters: mem -t 4 -k 32 -M), and the mapping rate and coverage were counted according to the alignment results. Duplicates were removed by SAMTOOLS [2].

**SNP detection and annotation**

The individual SNP variations were detected using SAMTOOLS [2] with the following parameter: 'mpileup -m 2 -F 0.002 -d 1000'. ANNOVAR [3] was used to perform annotation of the detected SNPs. To reduce the error rate in SNP detection, the results were filtered with the criterion as follows: (1) the number of support reads for each SNP should be more than 4 and (2) the mapping quality (MQ) of each SNP should be higher than 20.

**InDel detection and annotation**

SAMTOOLS [2] was used to detect InDels (insertion or deletion of ≤ 50 bp sequences in the DNA) with the following parameter 'mpileup -m 2 -F 0.002 -d 1000' followed by annotation using ANNOVAR [3]. The filter conditions were the same as with SNPs.

**SV detection and annotation**

BreakDancer [4] software were used to detect structural variants (SVs) including insertion (INS), deletion (DEL), inversion (INV), intra-chromosomal translocation (ITX) and inter-chromosomal translocation (CTX) mutations, based on the reference genome mapping results and the detected insert size. The detected SVs were filtered by removing those with less than 2 supporting PE reads. The INS, DEL and INV were further annotated by ANNOVAR. The Breakdancer software works with the read-pair method to detect the SVs.

**CNV detection and annotation**

Copy-number variation (CNV) based on the reads depth of the reference genome were used to detect CNVs of potential deletions and duplications with the following parameter '-call 100' using CNVnator [5]. The detected CNVs were further annotated by ANNOVAR [3].

**Visualization of variation**

For proper visualization of the structural variations on the whole-genome, they were presented according to mutation types with Circos [6]: (1) for SNP/InDel type, the density distribution and (2) for SV/CNV type, the location and size are drawn. The bioinformatic tools used can be viewed in Table S22.

**References**

1. Li H, Durbin R. Fast and accurate short read alignment with Burrows–Wheeler transform. Bioinformatics. 2009;25(14):1754-60; doi: 10.1093/bioinformatics/btp324.

2. Li H, Handsaker B, Wysoker A, Fennell T, Ruan J, Homer N, et al. The sequence alignment/map format and SAMtools. Bioinformatics. 2009;25(16):2078-9; doi: 10.1093/bioinformatics/btp352.

3. Wang K, Li M, Hakonarson H. ANNOVAR: functional annotation of genetic variants from high-throughput sequencing data. Nucleic Acids Research. 2010;38(16):e164-e; doi: 10.1093/nar/gkq603.

4. Chen K, Wallis JW, McLellan MD, Larson DE, Kalicki JM, Pohl CS, et al. BreakDancer: an algorithm for high-resolution mapping of genomic structural variation. Nature Methods. 2009;6(9):677-81; doi: 10.1038/nmeth.1363.

5. Abyzov A, Urban AE, Snyder M, Gerstein M. CNVnator: an approach to discover, genotype, and characterize typical and atypical CNVs from family and population genome sequencing. Genome Research. 2011;21(6):974-84.

6. Krzywinski M, Schein J, Birol İ, Connors J, Gascoyne R, Horsman D, et al. Circos: an information aesthetic for comparative genomics. Genome Research. 2009;19(9):1639-45; doi: 10.1101/gr.092759.109.
